# Identification of tumor antigens and immune landscapes for bladder urothelial carcinoma mRNA vaccine

**DOI:** 10.3389/fimmu.2023.1097472

**Published:** 2023-01-25

**Authors:** Zhuolun Sun, Changying Jing, Hailun Zhan, Xudong Guo, Ning Suo, Feng Kong, Wen Tao, Chutian Xiao, Daoyuan Hu, Hanbo Wang, Shaobo Jiang

**Affiliations:** ^1^ Department of Urology, The Third Affiliated Hospital of Sun Yat-sen University, Guangzhou, China; ^2^ Faculty of Medicine, Ludwig Maximilian University of Munich (LMU), Munich, Germany; ^3^ Institute of Diabetes and Regeneration, Helmholtz Zentrum München, German Research Center for Environmental Health, Neuherberg, Germany; ^4^ Department of Urology, Shandong Provincial Hospital Affiliated to Shandong First Medical University, Jinan, China; ^5^ Department of Urology, First Affiliated Hospital of Guangzhou Medical University, Guangzhou, China; ^6^ Department of Urology, The Sixth Affiliated Hospital of Sun Yat-sen University, Guangzhou, China

**Keywords:** bladder urothelial carcinoma, immune clusters, immune landscape, mRNA vaccine, tumor antigens

## Abstract

**Background:**

Bladder urothelial carcinoma (BLCA) is associated with high mortality and recurrence. Although mRNA-based vaccines are promising treatment strategies for combating multiple solid cancers, their efficacy against BLCA remains unclear. We aimed to identify potential effective antigens of BLCA for the development of mRNA-based vaccines and screen for immune clusters to select appropriate candidates for vaccination.

**Methods:**

Gene expression microarray data and clinical information were retrieved from The Cancer Genome Atlas and GSE32894, respectively. The mRNA splicing patterns were obtained from the SpliceSeq portal. The cBioPortal for Cancer Genomics was used to visualize genetic alteration profiles. Furthermore, nonsense-mediated mRNA decay (NMD) analysis, correlation analysis, consensus clustering analysis, immune cell infiltration analysis, and weighted co-expression network analysis were conducted.

**Results:**

Six upregulated and mutated tumor antigens related to NMD, and infiltration of APCs were identified in patients with BLCA, including HP1BP3, OSBPL9, SSH3, ZCCHC8, FANCI, and EIF4A2. The patients were subdivided into two immune clusters (IC1 and IC2) with distinct clinical, cellular and molecular features. Patients in IC1 represented immunologically ‘hot’ phenotypes, whereas those in IC2 represented immunologically ‘cold’ phenotypes. Moreover, the survival rate was better in IC2 than in IC1, and the immune landscape of BLCA indicated significant inter-patient heterogeneity. Finally, CALD1, TGFB3, and ANXA6 were identified as key genes of BLCA through WGCNA analysis, and their mRNA expression levels were measured using qRT-PCR.

**Conclusion:**

HP1BP3, OSBPL9, SSH3, ZCCHC8, FANCI, and EIF4A2 were identified as potential antigens for developing mRNA-based vaccines against BLCA, and patients in IC2 might benefit more from vaccination.

## Introduction

Bladder cancer (BC) is one of the most prevalent cancers worldwide. An estimated 83,730 new BC cases and 17,200 BC-related deaths were reported in the United States of America in 2021 (1). Increasing evidence implicates that BC is a clinically and genetically heterogeneous disease that is characterized by poor therapeutic efficacy and rapid tumor progression (2–4). More than 90% of BC cases are histologically categorized as bladder urothelial carcinoma (BLCA), which can present as non-muscle-invasive (75%) and muscle-invasive (25%) BC (NMIBC and MIBC, respectively) (4). Although the 5-year survival rate is as high as 90%, patients with NMIBC often relapse and progress to MIBC. Patients with MIBC usually have a poor prognosis because of aggressive metastasis and delayed diagnosis (5, 6). In addition to surgery, platinum-based chemotherapy is the first-line treatment for advanced or metastatic BLCA, which may extend median overall survival (OS) by approximately 1 year with a limited response rate (7, 8). However, non-responsive patients may lose the opportunity to receive additional therapeutic intervention for tumor development. Immune checkpoint blockade has recently emerged as a valuable treatment option for MIBC; however, its clinical benefits are observed only in a small proportion of patients (9, 10). These studies highlight the need for novel therapeutic strategies that may improve the clinical outcomes of patients with BLCA.

In the context of the ongoing coronavirus disease 2019 (COVID-19) pandemic, development of vaccines has been recognized as the top priority of pharmaceutical and biotechnology industries worldwide (11, 12). Therapeutic cancer vaccines are designed to reprogram the immune system of patients, specifically cytotoxic T lymphocytes, to safely and efficiently eliminate cancer cells (13). Antigens used for developing cancer vaccines include whole tumor cells, peptides, viral vectors, dendritic cells, DNA or RNA (14). The significant technological innovation and development investment in the last decade have made mRNA an optimal vehicle to carry tumor-specific antigens (15). Furthermore, mRNA-based vaccines are promising strategies for cancer therapy owing to their high efficacy, rapid development capabilities, safe administration and low-cost manufacturing as compared with other vaccine types (15–17). Recent preclinical and clinical trials have verified the viability of mRNA vaccines encoding tumor-specific antigens to combat multiple cancers, including lung cancer (18), prostate cancer (19), melanoma (20) and other cancers (15). However, tumor-specific or tumor-associated antigens (TSAs or TAAs, respectively) vary greatly among individuals. Recognizing immunogenic tumor neoantigens and relieving inhibitory tumor microenvironment (TME) are the main obstacles to developing mRNA vaccines against BLCA (16).

Several studies have shown that disruption of transcriptional regulation at different stages can lead to the accumulation of a large number of abnormal transcripts in cancer cells (21). These aberrant transcripts usually harbor premature termination codon; even if they are transcribed, they may be subsequently degraded by an mRNA surveillance pathway termed nonsense-mediated mRNA decay (NMD) (22). A relationship between NMD and tumor immunity is frequently observed and recognized as an attractive target for cancer therapy in some cases (22, 23). Recent studies have demonstrated that transcripts that harbor aberrant splicing patterns and frameshift mutations express antigenic peptides, with the disruption of normal NMD functionality (24). Therefore, it is important to perform a comprehensive analysis of alternative splicing (AS) patterns and NMD for developing individualized mRNA vaccines against tumors.

In this study, we investigated the potential BLCA antigens for developing mRNA vaccines and elucidated the immune landscape to identify eligible patients for vaccination. We confirmed six tumor antigens relevant to NMD, AS and antigen-presenting cell infiltration and defined two immune clusters of patients with BLCA. The two immune clusters presented distinct clinical, molecular and tumor immune microenvironment (TIME) characteristics, which were consistent in TCGA and GSE32894 cohorts. In addition, we assessed the immune landscape of BLCA by analyzing the expression profile of immune-related genes in individual patients. Finally, we identified CALD1, TGFB3, and ANXA6 as key genes of BLCA through WGCNA analysis and measured their mRNA expression levels using qRT-PCR. Therefore, the present study provides information regarding the complicated TIME in patients with BLCA and offers a reliable reference for developing and administering cancer vaccines.

## Materials and methods

### Identification of tumor antigens

#### Data extraction

The RNA-sequencing data and clinical information of patients with BLCA were retrieved from The Cancer Genome Atlas (TCGA) (https://tcga-data.nci.nih.gov/tcga/) and Gene Expression Omnibus (GEO) (GSE32894, https://www.ncbi.nlm.nih.gov/geo/query/acc.cgi?acc=GSE32894). The inclusion criteria were as follows: (1) RNAs that were detectable in >30% of the samples and (2) OS time > 30 days. The detailed clinical characteristics of the patients enrolled in this study are summarized in **Supplementary Table 1**. The original gene IDs of the respective datasets were transformed into the corresponding gene symbols based on annotation information on the platform. In addition, the expression profiles were indicated as transcripts per millions for subsequent analyzes. The batch effects between different datasets were corrected using the ‘ComBat’ method.

#### Profiling of AS events

The mRNA splicing patterns of 18 healthy patients and 399 patients with BLCA were retrieved from TCGA SpliceSeq portal (https://bioinformatics.mdanderson.org/TCGASpliceSeq/). The percent spliced-in index (PSI) value, ranging from 0 to 1, is the ratio between reads including or excluding the designated exons and indicates the efficiency of certain splicing events (25). To improve the reliability of the results, the primary PSI data that contained vacancy values were removed. The overlapping sets between different AS events were visualized using UpSet plots drawn using the Upset R package (26). To determine cancer-associated AS events (CASEs) in BLCA, we compared the PSI values of AS events between normal and BLCA tissues, and the P-value was adjusted using the Benjamini–Hochberg (BH) method. AS events with an absolute log2 (fold change) ≥ 1 and an adjusted P-value < 0.05 were considered statistically significant.

#### cBioPortal analysis

The ‘maftools’ R package and cBioPortal for Cancer Genomics (cBioPortal, https://www.cbioportal.org/) were used to retrieve the mutation data from TCGA database to compare and visualize potential genetic variations in each sample (27). Statistical significance was defined as P-value < 0.05.

#### NMD analysis

We identified genes with abnormally upregulated AS events and frameshift mutations as candidate antigens against BLCA. Many studies have highlighted the relationship between NMD and tumor immunity and revealed the potential of NMD as a therapeutic target for cancers in some cases (24). Further NMD analysis may assist in developing individualized tumor vaccines, such as for melanoma (23). Patients with BLCA were divided into the low- or high-expression groups according to the median expression of NMD factors (UPF1, UPF2, UPF3A and UPF3B). Subsequently, the expression levels and AS events of candidate genes between the two groups were analyzed. P-value was calculated using the ‘ggpubr’ package (stat_compare_means function), with P-value < 0.05 as the threshold.

#### TIMER analysis

The Tumor Immune Estimation Resource (TIMER, https://cistrome.shinyapps.io/timer/) is a public online database that allows systematic evaluation of the immune infiltration data for different cancers from TCGA (28). In this study, TIMER was used to assess and demonstrate the Spearman correlation between the abundance of tumor-infiltrating immune cells (TIICs) and the expression of tumor antigens. Purity adjustments were performed using Spearman’s correlation analysis. Statistical significance was defined as P-value < 0.05.

#### Prediction of the peptides of antigens for BLCA samples

The Cancer Immunome Atlas (TCIA, https://tcia.at/home) was used to screen for peptides of neoantigens for each BLCA sample with default parameters. A list of peptides was obtained by selecting the ‘Neoantigens’ tab after inputting candidate genes for antigens in the TCIA filter.

### Identification of immune clusters

#### Immune-related gene data extraction

A total of 1894 immune-related genes (IRGs) were retrieved from The Immunology Database and Analysis Portal (ImmPort, https://www.immport.org/shared/home) (29) and a study of Charoentong et al. (30) for both discovery (TCGA) and validation (GSE32894) cohorts. We choose this data matrix as the validation cohort because it represents one of the most comprehensive datasets, including the most survival data as well as clinical stage and tumor grade. After filtering these candidate IRGs associated with prognosis, 233 prognostic genes in 399 BLCA samples and 371 prognostic genes in 224 BLCA samples were identified in the discovery and validations cohort, respectively.

#### Identification and validation of immune clusters

Consensus clustering was performed to determine robust immune clusters according to the expression profiles of 233 prognostic IRGs using the ‘ConsensusClusterPlus’ package. Specifically, the algorithm of partition around medoids was used for 500 bootstraps, with 80% patients being resampled and ‘1-Pearson correlation’ as the distance metric in TCGA cohort. The cluster number was tested from 2 to 9, and the optimal one was identified to yield the least ambiguous cluster assignments across clustering permutations and the most stable consensus matrix. The immune clusters were further confirmed in the GSE32894 cohort using similar settings. The coherence of the identified immune clusters was quantified in the two cohorts *via* in-group proportion and Spearman’s correlation analyzes. The prognostic significance of these immune clusters in the discovery cohort was estimated *via* Kaplan–Meier survival analysis and validated in the validation cohort. Clinical features of these immune clusters, including stage, grade, clinical T stage and sex, were assessed using the ‘ggplot2’ R package.

#### Molecular, cellular and immunological features of the BLCA immune clusters

Tumor mutation burden (TMB) and mutated gene counts were visualized among the BLCA immune subtypes using the ‘maftools’ R package. In addition, copy number variations (CNVs) were compared. The correlation of the immune clusters with immune checkpoints (ICPs) and NMD factors was analyzed using the Wilcoxon test. Multiple biomarkers have been identified to predict the prognosis of BLCA. Therefore, the association between different BLCA biomarkers from The Cancer Genome Interpreter (CGI, https://www.cancergenomeinterpreter.org/home) and the immune clusters was assessed (31). The anticancer immune activity of the immune clusters was estimated using the Tracking Tumor Immunophenotyping (TIP, http://biocc.hrbmu.edu.cn/TIP/) (32). Furthermore, the TME-based ESTIMATE approach was used to compute the immune scores of the immune clusters, and the ‘CIBERSORT’ R package was used to compare the infiltration of immune cells.

#### Immune landscape analysis

To further reveal the distribution of immune clusters in each patient, graph learning-based dimensionality reduction analysis was performed using gene expression data. The maximum number of components was set to 4. Moreover, an approach used by Mao et al. (33) was adopted for dimensionality reduction using the Discriminative Dimension Reduction Tree algorithm and the reduceDimension function of the ‘Monocle’ package. The immune landscape was demonstrated using the function plot cell trajectory, and the plots corresponding to different immune clusters were represented in different colors. In addition, Pearson correlation analysis was used to examine the correlation among 22 TIICs in individual principal components, and differences in the abundance of TIICs between clusters were analyzed using the Wilcoxon test.

#### Weighted gene co−expression network analysis

Prognostic IRGs were used to perform weighted co-expression network analysis (WGCNA) to obtain gene co-expression modules using the ‘WGCNA’ package (34). The soft-thresholding power was selected according to the scale-free network topology criterion to construct a correlation adjacency matrix. The resulting modules were used to estimate module eigengenes (MEs) and quantify module similarity. Univariate Cox regression analysis was performed to identify modules that were remarkably associated with patient survival (P < 0.05). Furthermore, Kyoto Encyclopedia of Genes and Genomes (KEGG) enrichment analysis was performed for genes in each module to annotate gene functions and pathways using the ‘clusterProfiler’ package (35). Module membership (MM) shows the correlation between genes and modules, and genes with MM > 0.85 were defined as hub genes in the prognostic modules.

#### Quantitative validation of hub genes using quantitative real-time polymerase chain reaction (qRC-PCR)

To validate hub gene expression levels measured by the microarray, the qRT-PCR analyzes were applied using Applied Biosystems 7500 Fast Real-Time PCR System (Thermo Fisher Scientific) with SYBR Premix Ex TaqTM kit (Takara, Dalian, China). Total RNA was isolated from 40 pairs of BLCA and tumor-adjacent normal tissues using TRIzol^®^ reagent (Invitrogen; Thermo Fisher Scientific, Inc.). Reactions were performed at 50 °C for 5 s (1 cycle) 95 °C for 15 min (1 cycle), followed by 95 °C for 15 s and 60 °C for 1 min (40 cycles). Each sample was run in triplicate. Relative mRNA levels were normalized against GAPDH. Data were analyzed using the 2^−ΔΔCq^ method. The primer sequences were listed in **Supplementary Table 2**.

## Results

### Identification of potential tumor antigens of BLCA

**Figure 1:**
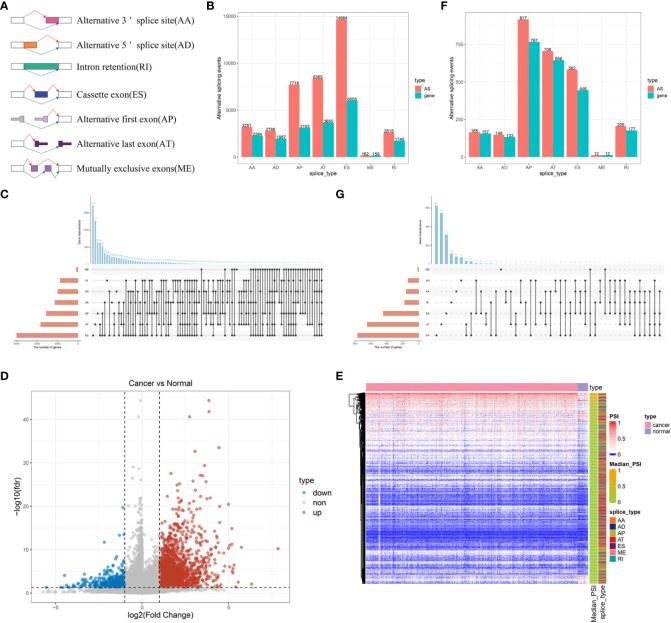
Profiling of integrated AS events detected in BLCA. **(A)** Schematic representation of seven different AS events. **(B)** The total number of AS events and the corresponding genes for each AS event in BLCA. **(C)** UpSet plot of interactive genes among seven different types of AS events. **(D)** Heatmap of CASEs between BLCA and normal tissues (|log2FC| ≥ 1, adjusted P < 0.05). **(E)** Volcano plot of CASEs identified in BLCA. **(F)** The total number of CASEs and the corresponding genes for each AS event in BLCA. **(G)** UpSet plot of interactive genes among seven different types of CASEs.

To identify potential antigens of BLCA, we first screened out aberrant AS events and overexpressed genes that could express TAAs. An integrated profile of AS events was established using the RNA-seq data of patients retrieved from TCGA database. Initially, 39,508 AS events were detected from 18,888 genes, accounting for approximately 92.78% of the potential protein-coding genes (36). The AS events are divided into seven types according to the splicing patterns, including alternate acceptor site (AA), alternate donor site (AD), retained intron (RI), exon skipping (ES), alternate promoter (AP), alternate terminator (AT) and mutually exclusive exons (ME) (**Figure 1A**). Among these splicing events, ES was the most predominant pattern identified, whereas ME was the least predominant (**Figure 1B**). Given that a single gene may have multiple AS events, an UpSet plot was generated to visualize the intersecting genes of each AS type. We found that ME always occurred in conjunction with other AS events in most cases, whereas PTK2 had all seven AS events (**Figure 1C**). To screen for BLCA-specific AS events, we conducted differential expression analysis by comparing 399 BLCA samples with 18 normal samples and identified 2736 CASEs (**Figures 1D, E**). Among these CASEs, 2352 were upregulated in 1776 genes, whereas 384 were downregulated in 340 genes (**Figure 1F**). Although ES was the predominant pattern, AP accounted for the highest proportion of CASEs, followed by AT (**Figure 1G**). The inconsonant distribution patterns among all AS events and CASEs suggested that each AS event played a distinct role in BLCA carcinogenesis.

Furthermore, we analyzed the mutation landscape of BLCA samples from TCGA and found that TP53 had the highest mutation rate (49%) (**Supplementary Figure 1A**). Tumor genomic mutations contribute to the appearance of neoantigens, and frameshift-mutation-derived peptides have been reported to have the highest immunogenicity (37). A total of 1451 genes with frameshift mutations encoding TSAs or TAAs were screened by evaluating fractional genomic alterations (**Supplementary Figure 1B**) and mutation counts (**Supplementary Figure 1C**) in patients, and TTN, TP53, MUC16, KMT2D, ARID1A, KDM6A, SYNE1, PIK3CA, KMT2C and RB1 were identified as the most frequent genetic mutations according to fractional genomic alterations (**Supplementary Figure 1D**) and mutation counts (**Supplementary Figure 1E**). This finding was consistent with the overall landscape of mutations. In addition, these 10 genes had the highest mutation count, suggesting underlying genomic interactions. Therefore, based on the combined analysis of the expression and mutation data of patients with BLCA, 153 overexpressed genes with frameshift mutations were identified as potential candidate antigens.

### Identification of tumor antigens associated with NMD and antigen-presenting cells

**Figure 2:**
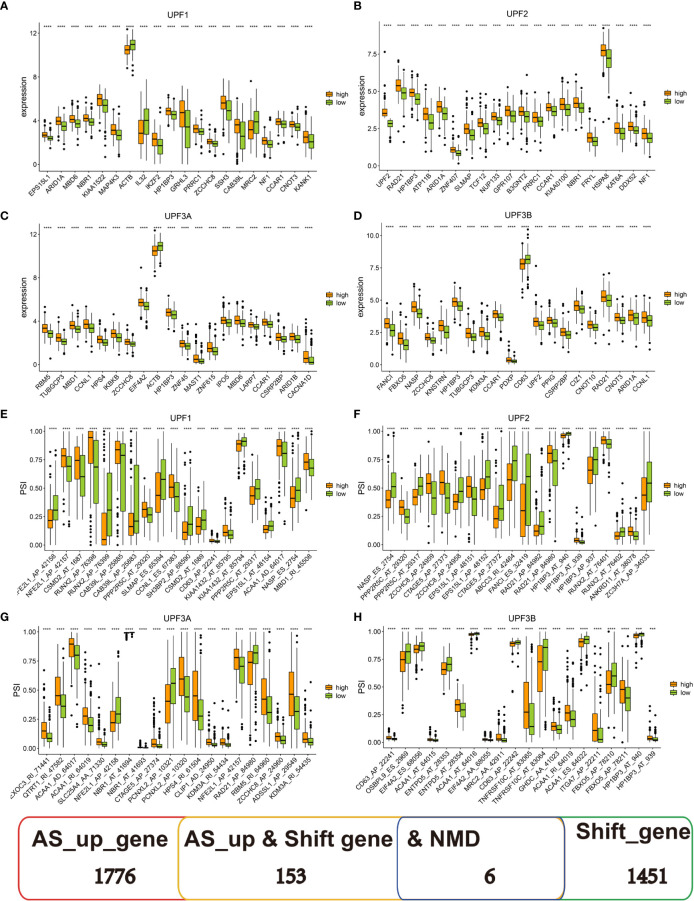
Identification of tumor antigens associated with nonsense-mediated mRNA decay factors. **(A–D)** The top 20 differentially expressed genes in four groups, including **(A)** UPF1, **(B)** UPF2, **(C)** UPF3A, and **(D)** UPF3B. **(E–H)** The top 20 CASEs in four groups, including **(E)** UPF1, **(F)** UPF2, **(G)** UPF3A and **(H)** UPF3B. *** P < 0.001, **** P < 0.0001.

Recently, transcripts harboring frameshift mutation and abnormal AS patterns have been reported to produce antigenic peptides by regulating NMD, which is a determinant of the efficacy of cancer immunotherapy (38). NMD-associated tumor antigens were selected from the identified genes as latent targets for mRNA vaccine development by analyzing the AS events and mutation landscape. We screened for differentially expressed genes in four groups: UPF1, UPF2, UPF3A and UPF3B (**Figures 2A–D**). The results revealed that most of the top 20 genes were significantly positively correlated with NMD expression in each group. In addition, we analyzed differences in the PSI value of 885 CASEs from 153 genes in the four NMD groups and found that the PSI values of a majority of CASEs were significantly higher in the high-NMD-expression group than in the low-NMD-expression group. The top 20 CASEs among four groups are shown in **Figure 2E–H**. Finally, six potential antigens, namely, HP1BP3, OSBPL9, SSH3, ZCCHC8, FANCI and EIF4A2, were identified through the intersection of overexpressed genes, genes with frameshift mutations and NMD-related genes (**Supplementary Figure 2**). Analysis of immune cell infiltration demonstrated that elevated expression of HP1BP3, OSBPL9, ZCCHC8, FANCI and EIF4A2 was associated with enhanced infiltration of B cells, macrophages and/or DCs **(Supplementary Figures 3A–E**). In addition, high SSH3 expression was associated with the infiltration of immune cells with some fluctuant (**Supplementary Figure 3F**). These results suggest that the six neoantigens produced during oncogenesis can be processed and presented by APCs, leading to the initiation of immune responses, and hence are promising targets for developing mRNA vaccines against BLCA with underlying immune activation functions. The peptides of six neoantigens predicted based on TCIA data are listed in **Supplementary Table 3**.

### Identification of potential immune clusters of BLCA

**Figure 3:**
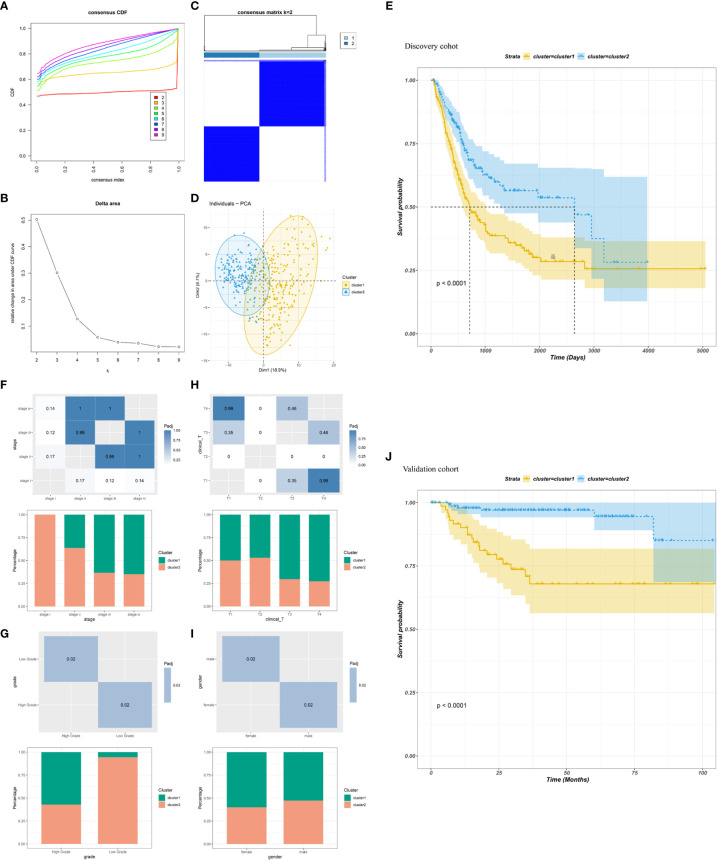
Identification of immune clusters of BLCA. **(A)** Cumulative distribution function curve and **(B)** delta area of immune-related genes in TCGA cohort. **(C)** Sample clustering heatmap. **(D)** Principal component analysis demonstrating two distinct clusters reflecting immune status. **(E)** Survival analysis of BLCA immune clusters in TCGA cohort. **(F–I)** Distribution of IC1–2 based on **(F)** stage, **(G)** grade, **(H)** clinical T stage and **(I)** sex in TCGA cohort. **(J)** Survival analysis of BLCA immune clusters in the GSE32894 cohort.

The heterogeneity of TME poses a challenge to cancer immunotherapy, especially in BLCA (39). Therefore, systematic investigation of immunotyping is of great importance to differentiate among patients with BLCA with diverse TIME, which may help in selecting eligible patients for vaccination. In this study, the expression profiles of 1894 IRGs in patients with BLCA were retrieved from TCGA database, and 233 IRGs were identified to be associated with prognosis and used to perform consensus clustering analysis. Based on the consensus accumulative distribution function and delta area (**Figures 3A, B**), we determined k as 2 for stable clustering of IRGs and obtained two immune clusters designated as IC1 and IC2 (**Figure 3C**). Principal component analysis revealed that patients in the two clusters were distributed in different directions (**Figure 3D**). In addition, survival was different between the two clusters; patients in IC1 had a poor prognosis (**Figure 3E**). Subtype distribution across different clinicopathological features revealed that patients with different stages, grades and clinical T stages were regularly clustered (**Figures 3F–H**). However, the sex of patients was unsuitable for further differentiation because sex distribution between the two clusters was similar (**Figure 3I**). The results obtained in TCGA cohort were validated in the GSE32894 cohort using the same approach, and 224 patients with BLCA were divided into two immune clusters (**Supplementary Figures 4A–D**). We then compared the distribution of different clinicopathological features in two clusters in the GSE32894 cohort (**Supplementary Figures 4E–G**). These immune clusters also had significant differences in survival, and patients in IC1 had a poorer OS (**Figure 3J**), suggesting the stability and reproducibility of the established immune clusters. Therefore, these immune clusters can be used as effective prognostic biomarkers for BLCA and are superior to conventional clinical indicators.

### Association of immune clusters with mutation status

**Figure 4:**
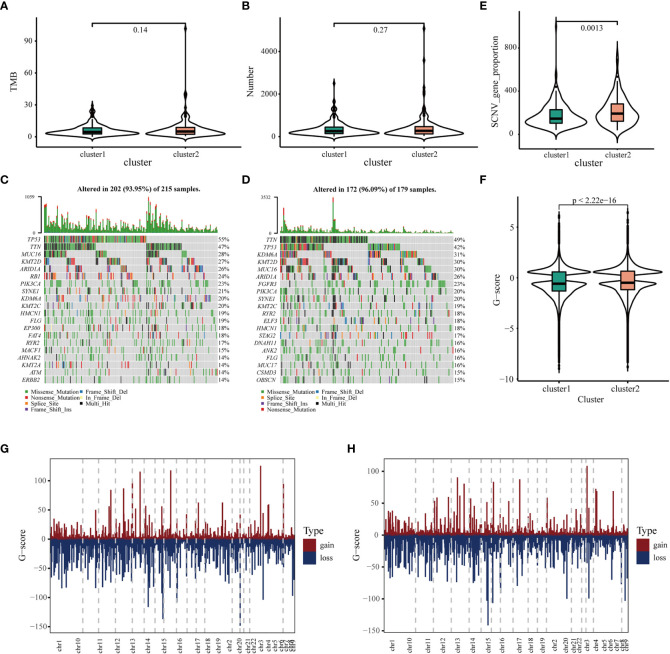
Association of immune clusters with mutation. **(A)** Tumor mutation burden and **(B)** mutation count across IC1 and IC2. Waterfall diagram of top 20 mutated genes in **(C)** IC1 and **(D)** IC2. **(E)** Association of immune clusters with somatic CNVs. **(E, F)** Association of immune clusters with G-score. **(G, H)** Gain or loss frequencies of CNVs across chromosomes in **(G)** IC1 and **(H)** IC2.

It has been reported that TMB and somatic mutation rates can be used to evaluate immunotherapeutic efficacy (40). In this study, TMB and mutations were calculated in the two clusters using the mutation data retrieved from TCGA database. No differences were observed in TMB and the number of mutated genes between the two clusters (**Figures 4A, B**). After analyzing the distribution of the top 20 mutations between two immune clusters, we found that TMB was less extensive in IC1 than in IC2 (93.95 versus 96.09%, respectively). TP53 mutation was significantly more frequent in CI1 than in CI2; however, contradictory results were observed regarding the mutation levels of TTN, MUC16 and KMT2D (**Figure 4C, D**). It has been reported that copy number alterations (CNAs) are one of the most important hallmarks of the progression of malignancies (41). We found that the frequency of somatic CNVs was significantly lower in patients in IC1 than in patients in IC2 (**Figure 4E**). In addition, the GISTIC score (G-score) of each patient was evaluated, with an absolute value greater than the threshold of 0.4 based on TCGA data. We found that the G-score varied markedly between the two clusters and was higher in IC2 (**Figure 4F**). The distribution of CNVs, with either deletions or gains, across all chromosomes was also assessed in the two clusters (**Figures 4G, H**). These results suggested that the immune clusters could assess the TMB, somatic mutation rates and CNAs of patients with BLCA to a certain extent, which may provide a basis for development of vaccines in the future.

### Association of immune clusters with ICPs and NMD factors

ICPs and NMD factors play an important role in anti-tumor immunity, which may affect the response to mRNA vaccines (9, 23). Therefore, we further examined the expression patterns of ICPs and NMD factors in different clusters. A total of 43 ICP-related modulators were detected in TCGA cohort; of which 38 (88.4%) exhibited significant differences between immune clusters (**Supplementary Figure 5A**). Among these 38 differentially expressed ICP-related genes, only three (TNFRSF14, TNFRSF25 and TNFSF15) were downregulated, whereas almost all other genes were upregulated in IC1. In addition, 36 (90.0%) out of 40 ICP-related genes were differentially expressed in the GSE32894 cohort, and all of them were upregulated in IC1 (**Supplementary Figure 4B**). Furthermore, four NMD factors were identified in both TCGA and GSE32894 cohorts. Two factors, namely, UPF1 and UPF3A, were diversely expressed in the two clusters in TCGA cohort and were upregulated in IC2 (**Supplementary Figure 5C**). Moreover, UPF3A expression was significantly different in the GSE32894 cohort and had the same expression pattern as that of TCGA cohort (**Supplementary Figure 5D**). Overall, the immune clusters mimicked the expression levels of ICPs and NMD factors, thus serving as potential biomarkers for predicting the efficacy of mRNA vaccines. mRNA vaccines may function better in IC2 owing to the relatively low expression of ICPs and high expression of NMD factors.

### Association of immune clusters with tumor markers

We systematically identified 16 prognostic and diagnostic markers of BLCA based on the CGI database. Of these genes, 10 had significantly different expressions between the two immune clusters in both TGCA and GSE32894 cohorts. The expression of CD274, FANCC and TUBB3 was significantly higher in IC1 than in IC2, whereas that of ERBB2, ERBB3, ERCC2, FGFR3, TP53, TSC1 and TSC2 was lower (**Supplementary Figures 6A, B**). However, these 10 markers have not been approved by the FDA and are either undergoing investigation in early trials or pre-clinical studies; therefore, their clinical applicability remains to be investigated. Currently, nuclear matrix protein 22 (NMP-22) is the most frequently used prognostic marker for BLCA, and patients with high expression have a significantly poorer prognosis (42). Therefore, we analyzed the expression of NMP-22 in patients with BLCA. Serum NMP-22 in IC2 in the TCGA cohort was significantly upregulated (**Supplementary Figure 6C**), while there was no significant difference in GSE32894 between the two clusters (**Supplementary Figure 6D**). Overall, the results revealed that the immune clusters were superior to other currently available cancer biomarkers in predicting patient outcomes.

### Association of immune clusters with immune microenvironment characteristics

**Figure 5:**
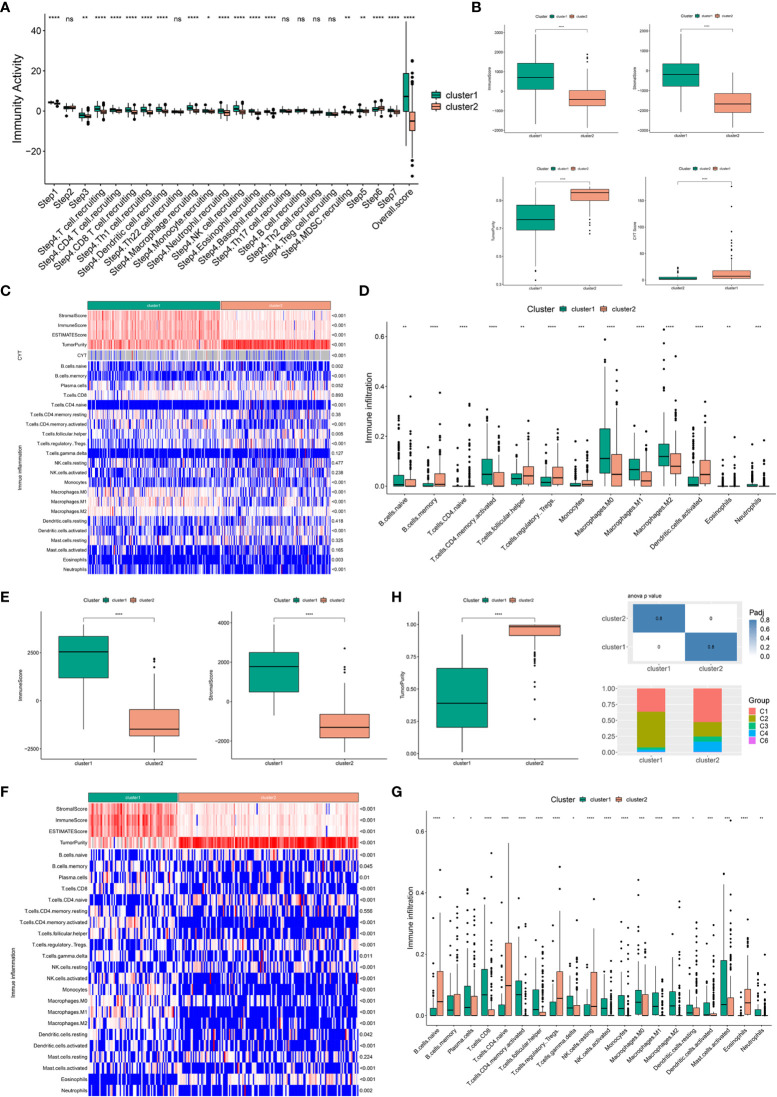
Association of immune clusters with immune microenvironment characteristics. **(A)** Distribution of immune activity scores in IC1 and IC2. **(B)** Association of immune subtypes with immune scores, stromal scores, tumor purity and CYT in TCGA cohort. Heatmap **(C)** and bar plot **(D)** of the relationship between immune clusters and immune cell subpopulations in TCGA cohort. **(E)** Association of immune subtypes with immune scores, stromal scores and tumor purity in the GSE32894 cohort. Heatmap **(F)** and bar plot **(G)** of the relationship between immune clusters and immune cell subpopulations in the GSE32894 cohort. **(H)** Distribution of individual immune categories in the two immune clusters. *P < 0.05, **P < 0.01, ***P < 0.001, ****P < 0.0001; ns, not significant.

Considering that the efficacy of mRNA vaccines is greatly associated with the immune status tumors, immune activity scores were first assessed using the TIP approach for analyzing and visualizing the status of anti-cancer immunity in the two immune clusters using RNA-seq data of patients with BLCA retrieved from TCGA. The overall score differed significantly between the two clusters, with patients in IC1 having a higher abundance of antitumor immune cells (**Figure 5A**). To further confirm the feasibility of clustering, we used the ESTIMATE algorithm to assess the immune features of BLCA in both TCGA and GSE32894 cohorts according to the expression of immune cell components. We found that patients in IC1 had higher stromal, immune and ESTIMATE scores but lower tumor purity and cytolytic activity (CYT) (**Figure 5B**). These results are consistent with those of previous studies, which have reported that low tumor purity (43) and CYT (44) serve as robust indicators for unfavorable prognosis. Furthermore, we examined differences in the abundance of 22 TIICs between the two clusters and found higher enrichment scores in IC1 (**Figure 5C**). For example, patients in IC1 had higher infiltration of naive B cells, activated memory CD4 T cells, macrophages and neutrophils (**Figure 5D**). Subsequent analyzes in the GSE32894 cohort yielded similar results (**Figures 5E–G**). Moreover, patients in IC1 had significantly higher infiltration of a majority of immune cells, including but not limited to memory B cells, plasma cells, CD8 T cells and activated memory CD4 T cells. Therefore, IC1 was considered an immunologically ‘hot’ phenotype, whereas IC2 was considered an immunologically ‘cold’ phenotype. Based on the abovementioned analyzes, we speculated that immune clusters can evaluate the immune status of BLCA and may help in selecting eligible patients for mRNA vaccination. These vaccines may be involved in the activation of various TIICs in immunologically ‘cold’ IC2. The six pan-cancer immune categories (C1–C6) defined by Thorsson et al. were closely related to prognosis and immunoregulation in tumors (45). As shown in **Figure 5H**, a distinct distribution of C1–C6 was observed in IC1 and IC2. In addition, there was a large degree of overlap in the proportion of C1–2 between the two immune clusters. The proportion of C1, C3 and C4 increased significantly, whereas that of IC2 decreased significantly in IC2 as compared to IC1. Patients in IC2 with a longer survival duration may be associated with the high proportion of C3 samples in IC2. These results facilitated a deeper understanding of the characteristics of the immune microenvironment in BLCA while further complementing previous studies.

### Immune landscape of BLCA

**Figure 6:**
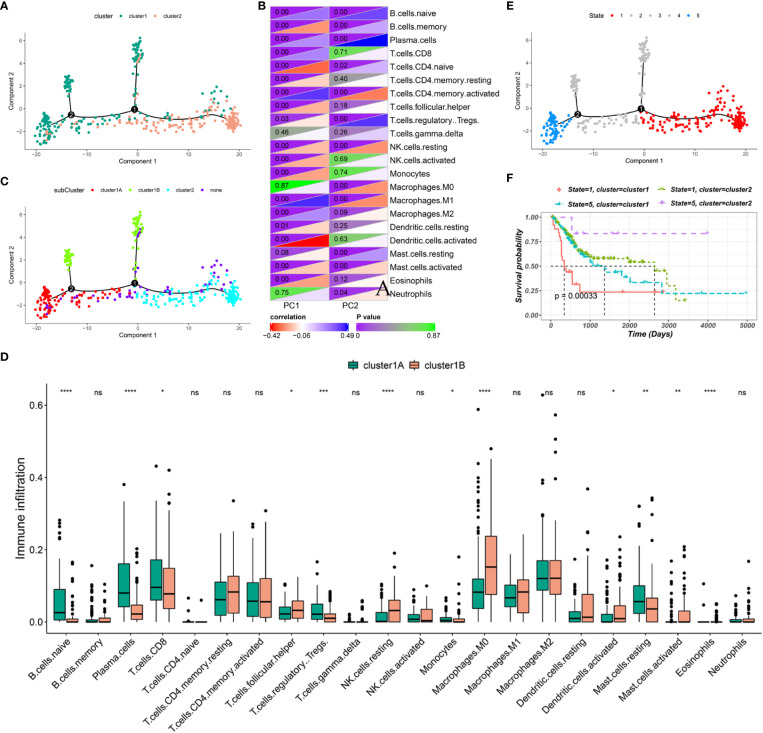
Immune landscape of BLCA. **(A)** Immune landscape of BLCA. Each point represents a patient, and the immune clusters are color-coded. **(B)** Correlation between two principal components and immune cells. **(C)** Immune landscape of the subclusters of BLCA immune clusters. **(D)** The proportion of certain immune cells in the IC1A–B subclusters. **(E)** Immune landscape of samples from five extreme locations and **(F)** their prognoses. *P < 0.05, **P < 0.01, ***P < 0.001, ****P < 0.0001; ns, not significant.

The immune gene expression profiles were integrated to assess the immune landscape of BLCA (**Figure 6A**). We found that the overall pattern of IC1 and IC2 distribution was reversed in the immune landscape. Principal component 1 (horizontal axis) had a positive correlation with activated memory CD4 T cells and M1 macrophages but a negative correlation with activated dendritic cells and naive CD4 T cells. In addition, principal component 2 (vertical axis) was most positively correlated with plasma cells and Tregs but most negatively correlated with activated memory CD4 T cells, resting NK cells and M1 macrophages (**Figure 6B**). The correlations among different immune cells between two principal components further demonstrated the accuracy of our approach. Heterogeneity can be found within the same cluster, and IC1 presented opposing distribution. Therefore, we stratified IC1 into two subclusters (IC1A and IC1B) based on the distribution of immune cell populations (**Figure 6C**). Considerable differences in the proportion of certain immune cells were observed. IC1B had a higher enrichment score of M0 macrophages and activated dendritic cells and a lower enrichment score of CD8 T cells (**Figure 6D**), suggesting that mRNA vaccines may be more effective in IC1B. In addition, we conducted the survival analysis of extremely distributed samples in the immune landscape and found that the survival rate of state 5 was significantly higher than that of state 1, suggesting that immune cluster-based immune landscape can be used to assess patient outcomes (**Figures 6E, F**). Collectively, the immune landscape based on immune clusters precisely identified immune components in each patient and predicted their outcomes, thus facilitating individualized mRNA vaccination.

### Identification of immune gene co-expression modules and hub genes of BLCA

**Figure 7:**
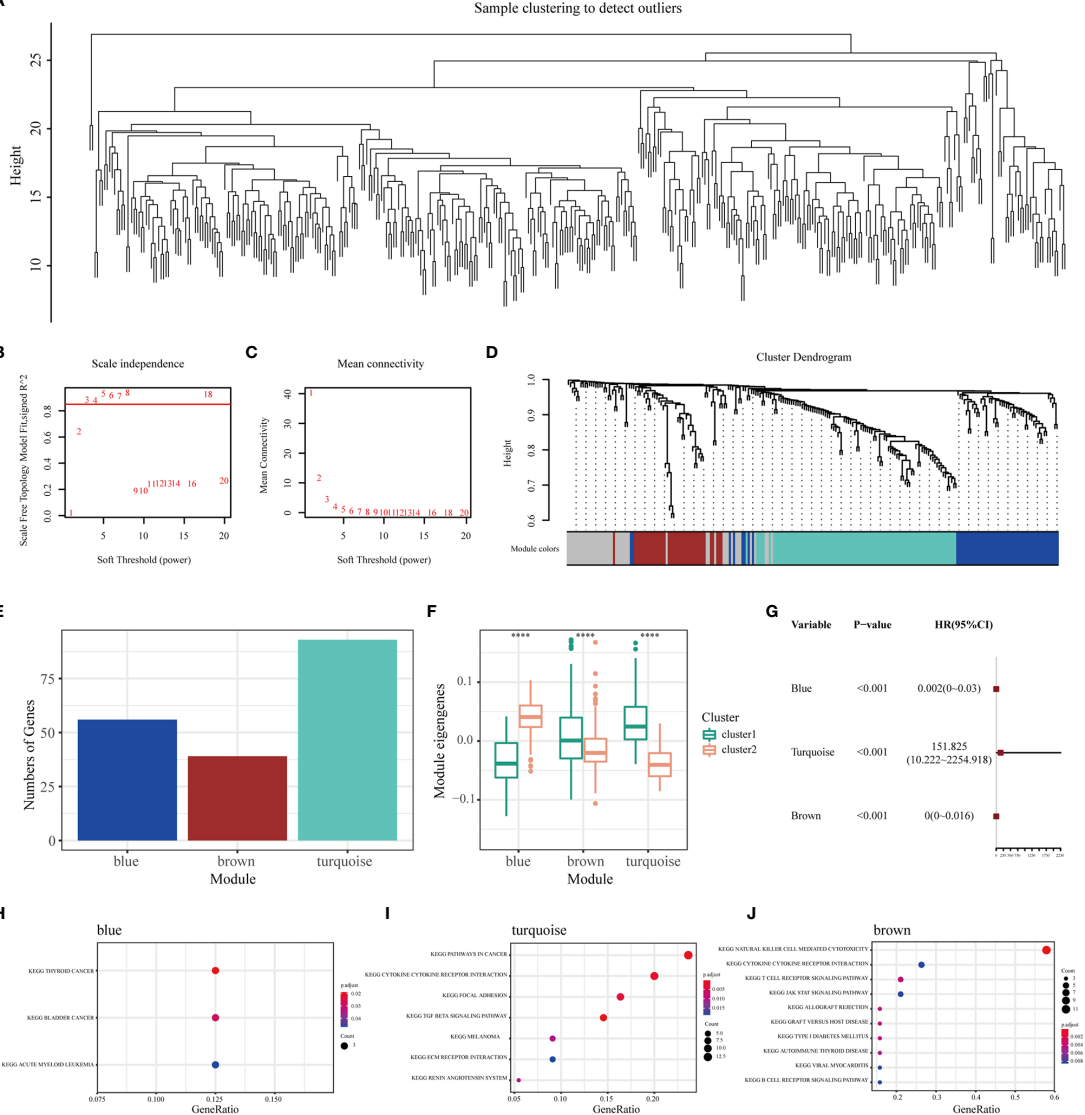
Identification of immune gene co-expression modules and hub genes of BLCA. **(A)** Sample clustering. **(B)** Scale-free fit index and **(C)** mean connectivity for various soft-thresholding powers. **(D)** Dendrogram of immune genes clustered based on the average-linkage hierarchy clustering method. **(E)** The number of genes in each module. **(F)** Differential distribution of MEs of each module in BLCA immune clusters. **(G)** Prognostic analysis of the blue, turquoise and brown modules. The top KEGG terms enriched in the **(H)** blue, **(I)** turquoise and **(J)** brown modules. ****P < 0.0001.

WGCNA was performed to identify immune gene co-expression modules containing immune genes associated with the effectiveness of mRNA vaccines. No outliers were found in the sample clustering (**Figure 7A**), and a soft-threshold power of β = 3 (scale-free R2 = 0.85) was selected to ensure a scale-free network (**Figures 7B, C**). Subsequently, the representation matrix was converted to an adjacency matrix and then to a topological matrix. Considering the minimum module size of 30 genes as a criterion, a dendrogram was constructed using the average-linkage hierarchy clustering method. MEs were calculated by merging the closed modules with a deep split of 5 and a height of 0.2 (**Figure 7D**). Eventually, four modules that contained similar gene patterns were identified, and the grey module included genes that were not present in any module (**Figure 7E**). We further examined MEs in the two immune clusters and noticed significantly different distribution of all three modules (except the grey module). IC1 had a higher number of MEs in the brown and turquoise modules, whereas IC2 showed higher eigengenes in the blue module (**Figure 7F**). Further prognostic correlation results suggested that the blue, turquoise and brown modules were distinctly associated with the prognosis of BLCA (**Figure 7G**). Moreover, functional enrichment analysis indicated that genes in the blue module were relevant to BC, those in the turquoise module were associated with pathways in cancer and those in the brown module were associated with natural killer cell-mediated cytotoxicity (**Figures 7H–J**). Eventually, three hub genes with relevance > 85% to MEs of three modules were identified, including CALD1, TGFB3 and ANXA6. These hub genes can be used as predictive and prognostic biomarkers and for identifying eligible patients with BLCA for mRNA vaccination. qRT-PCR was applied to examine the relative mRNA levels of CALD1, TGFB3 and ANXA6 in 40 pairs of BLCA and adjacent normal tissues. Results demonstrated that the expression levels of these 3 genes were higher in BLCA tissues than in tumor-adjacent normal tissues (**Supplementary Figure 7**).

## Discussion

BLCA is one of the most aggressive malignancies with significant tumor heterogeneity, multidrug resistance and uncontrolled metastasis (3, 5). Cisplatin combined with gemcitabine has been established as a standard therapeutic strategy for the management of patients with advanced BLCA; however, clinical benefits are limited (6, 7). Immunotherapy has revolutionized treatment paradigms in oncology, especially with the clinical success of immune checkpoint inhibitors; however, its effectiveness in BLCA remains unknown (10). mRNA-based vaccines with cancer antigens represent a promising alternative immunotherapeutic strategy, with multiple ongoing human clinical trials (15). A recent study demonstrated that a combination of mRNA vaccine and immune checkpoint inhibitor can enhance the immune response against melanoma and inhibit tumor progression (20). However, the efficacy of mRNA vaccines in patients with BLCA remains unknown.

In this study, we systematically profiled the aberrant AS events and mutational landscape of BLCA for future development of individualized mRNA-based cancer vaccines. To elucidate the clinical relevance of the selected antigens, their correlation with NMD factors and immune cell infiltration was examined. Six tumor antigens (HP1BP3, OSBPL9, SSH3, ZCCHC8, FANCI and EIF4A2) were correlated with the expression of NMD factors and infiltration of APCs, which may be promising candidates for mRNA vaccines. These findings revealed the importance of these candidates in the development of BLCA, which can be recognized and presented directly to T cell receptors to eradicate tumor cells and induce antitumor immunity. Although functional validation and clinical evaluation of these candidate genes require further investigation, previous studies have demonstrated their potential for developing mRNA vaccines against tumors. In recent years, several studies have focused on the role of EIF4A2 in regulating immune responses and numerous cellular and pathophysiological processes, which serves as a prognostic biomarker and is correlated with immune infiltration in multiple cancers, including BLCA (46, 47).

Because therapeutic effects of mRNA vaccines vary between individuals, patients with BLCA were divided into two immune clusters (IC1 and IC2) based on their immune-related gene profiles to identify eligible patients for vaccination. The two immune clusters possessed distinct clinical, molecular and TIME features. For example, patients in IC2 had a better prognosis in both TCGA and GSE32894 cohorts, suggesting that the immune cluster could serve as a prognostic biomarker for BLCA. In addition, we observed that the predictive power of IC2 was superior to conventional tumor biomarkers such as NMP-22. In addition, this cluster can be used to predict the response to vaccine therapy. Elevated rates of somatic CNVs in IC2 are suggestive of greater responsiveness to mRNA vaccines. With regard to the expression of ICPs and NMD factors, mRNA vaccines might have better efficacy in IC2. The immune activity of the two immune subtypes was assessed *via* TIP analysis. IC2 had strikingly lower immune activity, suggesting that mRNA vaccines targeting IC1 might reinforce its immune response.

Given that the tumor immune status is critical for the efficacy of mRNA vaccines, we further investigated the immune cell components and found a strikingly distinct TIME in the two immune clusters. This finding suggested that the two clusters might have varying mechanisms for regulating immune escape in tumors, which may require individualized therapeutic strategies. In addition, we found that IC2 had an immunologically cold phenotype with less infiltration of immune cells (an ‘immune desert’) and immunologically inactive ‘non-inflamed’ tumors. This phenotype may be related to the lack of APCs and tumor antigens, resulting in T cell anergy and insensitivity to antigen activation. To reinvigorate the immune system of such patients against tumor cells, mRNA-based vaccines that trigger immune cell infiltration may be an appropriate option. However, IC1 had a favorable immunologically hot phenotype, characterized by the increased infiltration of immune cells and immunologically active ‘inflamed’ tumors. Therefore, ICBs are especially advantageous for patients in IC1, which may further regulate the production of CD8+ T lymphocytes and the suppression of Tregs, inducing antitumor immunity (48, 49). Recent studies have highlighted the role of inflammation in tumorigenesis and tumor progression, revealing a close relationship between inflammation and BLCA (50, 51). An inflammatory phenotype with a high density of macrophages might, at least partially, lead to poor outcomes in patients in IC1 (51). Another important consideration in determining the prognosis is the preponderance of the immune-suppressive or -stimulatory environment. In a study, patients with BLCA were divided into C1-C6 subtypes, except for the C5 subtype, based on previous immunotyping studies among 33 cancer types (45). The C3 subtype was associated with the best prognosis, followed by the C1, C2, C4 and C6 subtypes. Our data showed substantial variations in the proportion of five categories in IC1 and IC2. Patients in IC2 with a longer survival duration may be associated with the high proportion of C3 samples in IC2. Moreover, the proportion of C2 (IFN-g dominant) in IC1 was significantly higher than that in IC2, whereas the proportion of C4 (lymphocyte failure) showed the opposite trend. This finding provides further verified the ‘hot’ phenotype of IC1 and the ‘cold’ phenotype of IC2. Therefore, mRNA vaccine administration in IC2 might stimulate the immune response, thus converting the ‘cold’ TME to ‘hot’ by increasing the infiltration of inflammatory immune cells (49). Therefore, our results are reliable and complement the classification schemes previously developed.

Furthermore, the complex immune landscape of BLCA demonstrated substantial heterogeneity among individuals and within the same immune subgroups, thus facilitating the accurate determination of immune cell components in each patient to aid in developing individualized mRNA vaccines. Intra-cluster heterogeneity observed in IC1 was based on the distribution of immune cell groups. The infiltration of M0 macrophages and activated dendritic cells was higher and that of CD8 T cells was lower in IC1B than in IC1A, suggesting that the therapeutic efficacy of mRNA vaccines may be better in patients in IC1B. In such patients, novel treatment strategies based on mRNA vaccines combined with chemotherapy or immunotherapy may modulate both TME and immune response of the host, which is considered more conducive to successful therapy (52).

Furthermore, we used WGCNA to construct co-expressed gene modules and identified three key modules (blue, turquoise and brown) significantly correlated with each immune cluster, which was of fundamental importance in investigating the underlying biological mechanisms of the clusters. Subsequent KEGG analysis suggested that the three modules had substantial disparity among the involved pathways, suggesting that the classification method had high discrimination power. In addition, CALD1, TGFB3 and ANXA6 were identified as immune hub genes (MM > 0.85), which may serve as biomarkers for predicting the outcomes of patients with BLCA and selecting eligible patients for mRNA vaccination.

Therefore, this study provides critical insights into developing mRNA vaccines for other diseases. The emergence of the COVID-19 pandemic made mRNA vaccines an innovative and promising platform (53). Although mRNA vaccines have protected millions of patients with COVID-19 and prevented many deaths worldwide, the evolving variants such as D614G require these vaccines to be updated periodically (54). Therefore, there are significant implications for improving the clinical treatment of COVID-19 by determining specific antigens and eligible patients for mRNA vaccine administration.

## Conclusion

In conclusion, HP1BP3, OSBPL9, SSH3, ZCCHC8, FANCI and EIF4A2 were identified as potential antigens for developing mRNA vaccines against BLCA. In addition, patients in IC2 may benefit more from mRNA vaccination. These findings provide new sights into developing mRNA vaccines against BLCA and defining the eligible population for mRNA vaccination.

## Data availability statement

Publicly available datasets were analyzed in this study, the names of the repositories/accession numbers are included within the article/**Supplementary Material**.

## Ethics statement

The studies involving human participants were reviewed and approved by Shandong Provincial Hospital Affiliated to Shandong First Medical University. The patients/participants provided their written informed consent to participate in this study.

## Author contributions

ZS, CJ, HZ and XG conceived the study, conducted bioinformatics analysis, and wrote the manuscript. NS and FK participated in drafting and revising the article. CX, WT, and DH refined the data analysis and prepared all the figures. HW and SJ revised and supervised the manuscript. All authors contributed to the article and approved the submitted version.

## References

[1] Siegel RL Miller KD Fuchs HE Jemal A. Cancer statistics, 2021. CA: Cancer J Clin (2021) 71(1):7–33. doi: 10.3322/caac.21654 33433946

[2] Robertson AG Kim J Al-Ahmadie H Bellmunt J Guo G Cherniack AD. Comprehensive molecular characterization of muscle-invasive bladder cancer. Cell (2017) 171(3):540–56.e25. doi: 10.1016/j.cell.2017.09.007 28988769 PMC5687509

[3] Knowles MA Hurst CD. Molecular biology of bladder cancer: New insights into pathogenesis and clinical diversity. Nat Rev Cancer (2015) 15(1):25–41. doi: 10.1038/nrc3817 25533674

[4] Cumberbatch MGK Jubber I Black PC Esperto F Figueroa JD Kamat AM. Epidemiology of bladder cancer: A systematic review and contemporary update of risk factors in 2018. Eur Urol (2018) 74(6):784–95. doi: 10.1016/j.eururo.2018.09.001 30268659

[5] Dy GW Gore JL Forouzanfar MH Naghavi M Fitzmaurice C. Global burden of urologic cancers, 1990-2013. Eur Urol (2017) 71(3):437–46. doi: 10.1016/j.eururo.2016.10.008 28029399

[6] Nadal R Bellmunt J. Management of metastatic bladder cancer. Cancer Treat Rev (2019) 76:10–21. doi: 10.1016/j.ctrv.2019.04.002 31030123

[7] Pietzak EJ Zabor EC Bagrodia A Armenia J Hu W Zehir A. Genomic differences between "Primary" and "Secondary" muscle-invasive bladder cancer as a basis for disparate outcomes to cisplatin-based neoadjuvant chemotherapy. Eur Urol (2019) 75(2):231–9. doi: 10.1016/j.eururo.2018.09.002 PMC6339572 30290956

[8] Huang HM Li HX. Tumor heterogeneity and the potential role of liquid biopsy in bladder cancer. Cancer Commun (London England) (2021) 41(2):91–108. doi: 10.1002/cac2.12129 PMC7896752 33377623

[9] Bellmunt J Powles T Vogelzang NJ. A review on the evolution of pd-1/Pd-L1 immunotherapy for bladder cancer: The future is now. Cancer Treat Rev (2017) 54:58–67. doi: 10.1016/j.ctrv.2017.01.007 28214651

[10] Chism DD. Urothelial carcinoma of the bladder and the rise of immunotherapy. J Natl Compr Cancer Network JNCCN (2017) 15(10):1277–84. doi: 10.6004/jnccn.2017.7036 28982752

[11] Wu S Zhong G Zhang J Shuai L Zhang Z Wen Z. A single dose of an adenovirus-vectored vaccine provides protection against sars-Cov-2 challenge. Nat Commun (2020) 11(1):4081–. doi: 10.1038/s41467-020-17972-1 PMC7427994 32796842

[12] Pushparajah D Jimenez S Wong S Alattas H Nafissi N Slavcev RA. Advances in gene-based vaccine platforms to address the covid-19 pandemic. Advanced Drug Del Rev (2021) 170:113–41. doi: 10.1016/j.addr.2021.01.003 PMC7789827 33422546

[13] Xia L Schrump DS Gildersleeve JC. Whole-cell cancer vaccines induce Large antibody responses to carbohydrates and glycoproteins. Cell Chem Biol (2016) 23(12):1515–25. doi: 10.1016/j.chembiol.2016.10.012 PMC5182097 27889407

[14] Huang X Zhang G Tang T Liang T. Identification of tumor antigens and immune subtypes of pancreatic adenocarcinoma for mrna vaccine development. Mol Cancer (2021) 20(1):44. doi: 10.1186/s12943-021-01310-0 33648511 PMC7917175

[15] Pardi N Hogan MJ Porter FW Weissman D. Mrna vaccines - a new era in vaccinology. Nat Rev Drug Discovery (2018) 17(4):261–79. doi: 10.1038/nrd.2017.243 PMC5906799 29326426

[16] Miao L Zhang Y Huang L. Mrna vaccine for cancer immunotherapy. Mol Cancer (2021) 20(1):41–. doi: 10.1186/s12943-021-01335-5 PMC7905014 33632261

[17] Van Hoecke L Verbeke R Dewitte H Lentacker I Vermaelen K Breckpot K. Mrna in cancer immunotherapy: Beyond a source of antigen. Mol Cancer (2021) 20(1):48–. doi: 10.1186/s12943-021-01329-3 PMC7926200 33658037

[18] Sebastian M Schröder A Scheel B Hong HS Muth A von Boehmer L. A phase I/Iia study of the mrna-based cancer immunotherapy Cv9201 in patients with stage Iiib/Iv non-small cell lung cancer. Cancer Immunol Immunother (2019) 68(5):799–812. doi: 10.1007/s00262-019-02315-x 30770959 PMC11028316

[19] Kübler H Scheel B Gnad-Vogt U Miller K Schultze-Seemann W Vom Dorp F. Self-adjuvanted mrna vaccination in advanced prostate cancer patients: A first-in-Man phase I/Iia study. J immunotherapy Cancer (2015) 3:26. doi: 10.1186/s40425-015-0068-y PMC4468959 26082837

[20] Wang Y Zhang L Xu Z Miao L Huang L. Mrna vaccine with antigen-specific checkpoint blockade induces an enhanced immune response against established melanoma. Mol Ther J Am Soc Gene Ther (2018) 26(2):420–34. doi: 10.1016/j.ymthe.2017.11.009 PMC5835019 29249397

[21] Suzuki A Makinoshima H Wakaguri H Esumi H Sugano S Kohno T. Aberrant transcriptional regulations in cancers: Genome, transcriptome and epigenome analysis of lung adenocarcinoma cell lines. Nucleic Acids Res (2014) 42(22):13557–72. doi: 10.1093/nar/gku885 PMC4267666 25378332

[22] Lindeboom RG Supek F Lehner B. The rules and impact of nonsense-mediated mrna decay in human cancers. Nat Genet (2016) 48(10):1112–8. doi: 10.1038/ng.3664 PMC5045715 27618451

[23] Litchfield K Reading JL Lim EL Xu H Liu P Al-Bakir M. Escape from nonsense-mediated decay associates with anti-tumor immunogenicity. Nat Commun (2020) 11(1):3800. doi: 10.1038/s41467-020-17526-5 32733040 PMC7393139

[24] Oka M Xu L Suzuki T Yoshikawa T Sakamoto H Uemura H. Aberrant splicing isoforms detected by full-length transcriptome sequencing as transcripts of potential neoantigens in non-small cell lung cancer. Genome Biol (2021) 22(1):9–. doi: 10.1186/s13059-020-02240-8 PMC7780684 33397462

[25] Schafer S Miao K Benson CC Heinig M Cook SA Hubner N. Alternative splicing signatures in rna-seq data: Percent spliced in (Psi). Curr Protoc Hum Genet (2015) 87:11. doi: 10.1002/0471142905.hg1116s87 26439713

[26] Conway JR Lex A Gehlenborg N. Upsetr: An r package for the visualization of intersecting sets and their properties. Bioinf (Oxford England) (2017) 33(18):2938–40. doi: 10.1093/bioinformatics/btx364 PMC5870712 28645171

[27] Cerami E Gao J Dogrusoz U Gross BE Sumer SO Aksoy BA. The cbio cancer genomics portal: An open platform for exploring multidimensional cancer genomics data. Cancer Discovery (2012) 2(5):401–4. doi: 10.1158/2159-8290.cd-12-0095 PMC3956037 22588877

[28] Li T Fan J Wang B Traugh N Chen Q Liu JS. Timer: A web server for comprehensive analysis of tumor-infiltrating immune cells. Cancer Res (2017) 77(21):e108–e10. doi: 10.1158/0008-5472.can-17-0307 PMC6042652 29092952

[29] Bhattacharya S Andorf S Gomes L Dunn P Schaefer H Pontius J. Immport: Disseminating data to the public for the future of immunology. Immunologic Res (2014) 58(2-3):234–9. doi: 10.1007/s12026-014-8516-1 24791905

[30] Charoentong P Finotello F Angelova M Mayer C Efremova M Rieder D. Pan-cancer immunogenomic analyses reveal genotype-immunophenotype relationships and predictors of response to checkpoint blockade. Cell Rep (2017) 18(1):248–62. doi: 10.1016/j.celrep.2016.12.019 28052254

[31] Tamborero D Rubio-Perez C Deu-Pons J Schroeder MP Vivancos A Rovira A. Cancer genome interpreter annotates the biological and clinical relevance of tumor alterations. Genome Med (2018) 10(1):25. doi: 10.1186/s13073-018-0531-8 29592813 PMC5875005

[32] Xu L Deng C Pang B Zhang X Liu W Liao G. Tip: A web server for resolving tumor immunophenotype profiling. Cancer Res (2018) 78(23):6575–80. doi: 10.1158/0008-5472.can-18-0689 30154154

[33] Qi M Li W Tsang IW Yijun S. Principal graph and structure learning based on reversed graph embedding. IEEE Trans Pattern Anal Mach Intell (2017) 39(11):2227–41. doi: 10.1109/tpami.2016.2635657 PMC5899072 28114001

[34] Langfelder P Horvath S. Wgcna: An r package for weighted correlation network analysis. BMC Bioinf (2008) 9:559. doi: 10.1186/1471-2105-9-559 PMC2631488 19114008

[35] Yu G Wang LG Han Y He QY. Clusterprofiler: An r package for comparing biological themes among gene clusters. Omics J Integr Biol (2012) 16(5):284–7. doi: 10.1089/omi.2011.0118 PMC3339379 22455463

[36] Pertea M Shumate A Pertea G Varabyou A Breitwieser FP Chang Y-C. Chess: A new human gene catalog curated from thousands of Large-scale rna sequencing experiments reveals extensive transcriptional noise. Genome Biol (2018) 19(1):208–. doi: 10.1186/s13059-018-1590-2 PMC6260756 30486838

[37] Giannakis M Mu XJ Shukla SA Qian ZR Cohen O Nishihara R. Genomic correlates of immune-cell infiltrates in colorectal carcinoma. Cell Rep (2016) 15(4):857–65. doi: 10.1016/j.celrep.2016.03.075 PMC4850357 27149842

[38] Lindeboom RGH Vermeulen M Lehner B Supek F. The impact of nonsense-mediated mrna decay on genetic disease, gene editing and cancer immunotherapy. Nat Genet (2019) 51(11):1645–51. doi: 10.1038/s41588-019-0517-5 PMC6858879 31659324

[39] Meeks JJ Al-Ahmadie H Faltas BM Taylor JA 3rd Flaig TW DeGraff DJ. Genomic heterogeneity in bladder cancer: Challenges and possible solutions to improve outcomes. Nat Rev Urol (2020) 17(5):259–70. doi: 10.1038/s41585-020-0304-1 PMC7968350 32235944

[40] Sha D Jin Z Budczies J Kluck K Stenzinger A Sinicrope FA. Tumor mutational burden as a predictive biomarker in solid tumors. Cancer Discovery (2020) 10(12):1808–25. doi: 10.1158/2159-8290.cd-20-0522 PMC7710563 33139244

[41] Chaluvally-Raghavan P Zhang F Pradeep S Hamilton MP Zhao X Rupaimoole R. Copy number gain of hsa-Mir-569 at 3q26.2 leads to loss of Tp53inp1 and aggressiveness of epithelial cancers. Cancer Cell (2014) 26(6):863–79. doi: 10.1016/j.ccell.2014.10.010 PMC4261159 25490449

[42] Chou R Gore JL Buckley D Fu R Gustafson K Griffin JC. Urinary biomarkers for diagnosis of bladder cancer: A systematic review and meta-analysis. Ann Internal Med (2015) 163(12):922–31. doi: 10.7326/m15-0997 26501851

[43] Zhang C Cheng W Ren X Wang Z Liu X Li G. Tumor purity as an underlying key factor in glioma. Clin Cancer Res an Off J Am Assoc Cancer Res (2017) 23(20):6279–91. doi: 10.1158/1078-0432.ccr-16-2598 28754819

[44] Rooney MS Shukla SA Wu CJ Getz G Hacohen N. Molecular and genetic properties of tumors associated with local immune cytolytic activity. Cell (2015) 160(1-2):48–61. doi: 10.1016/j.cell.2014.12.033 25594174 PMC4856474

[45] Thorsson V Gibbs DL Brown SD Wolf D Bortone DS Ou Yang TH. The immune landscape of cancer. Immunity (2018) 48(4):812–30.e14. doi: 10.1016/j.immuni.2018.03.023 29628290 PMC5982584

[46] De Benedetti A Graff JR. Eif-4e expression and its role in malignancies and metastases. Oncogene (2004) 23(18):3189–99. doi: 10.1038/sj.onc.1207545 15094768

[47] Crew JP Fuggle S Bicknell R Cranston DW de Benedetti A Harris AL. Eukaryotic initiation factor-4e in superficial and muscle invasive bladder cancer and its correlation with vascular endothelial growth factor expression and tumour progression. Br J Cancer (2000) 82(1):161–6. doi: 10.1054/bjoc.1999.0894 PMC2363195 10638984

[48] Sharpe AH. Introduction to checkpoint inhibitors and cancer immunotherapy. Immunol Rev (2017) 276(1):5–8. doi: 10.1111/imr.12531 28258698 PMC5362112

[49] Newman JH Chesson CB Herzog NL Bommareddy PK Aspromonte SM Pepe R. Intratumoral injection of the seasonal flu shot converts immunologically cold tumors to hot and serves as an immunotherapy for cancer. Proc Natl Acad Sci United States America (2020) 117(2):1119–28. doi: 10.1073/pnas.1904022116 PMC6969546 31888983

[50] Wigner P Grębowski R Bijak M Saluk-Bijak J Szemraj J. The interplay between oxidative stress, inflammation and angiogenesis in bladder cancer development. Int J Mol Sci (2021) 22(9):4483. doi: 10.3390/ijms22094483 33923108 PMC8123426

[51] Crispen PL Kusmartsev S. Mechanisms of immune evasion in bladder cancer. Cancer Immunol Immunother (2020) 69(1):3–14. doi: 10.1007/s00262-019-02443-4 31811337 PMC6949323

[52] Sahin U Oehm P Derhovanessian E Jabulowsky RA Vormehr M Gold M. An rna vaccine drives immunity in checkpoint-Inhibitor-Treated melanoma. Nature (2020) 585(7823):107–12. doi: 10.1038/s41586-020-2537-9 32728218

[53] Lazarus R Baos S Cappel-Porter H Carson-Stevens A Clout M Culliford L. Safety and immunogenicity of concomitant administration of covid-19 vaccines (Chadox1 or Bnt162b2) with seasonal influenza vaccines in adults in the uk (Comflucov): A multicentre, randomised, controlled, phase 4 trial. Lancet (London England) (2021) 398(10318):2277–87. doi: 10.1016/S0140-6736(21)02329-1 PMC8585490 34774197

[54] Weissman D Alameh MG de Silva T Collini P Hornsby H Brown R. D614g spike mutation increases sars cov-2 susceptibility to neutralization. Cell Host Microbe (2021) 29(1):23–31.e4. doi: 10.1016/j.chom.2020.11.012 33306985 PMC7707640

