# Supplementary material for: Identification of tumor antigens and immune landscapes for bladder urothelial carcinoma mRNA vaccine
Source: Front Immunol. 2023 Jan 25;14:1097472. doi: 10.3389/fimmu.2023.1097472 (PMC9905425; doi:10.3389/fimmu.2023.1097472)
Supplement: Supplementary file 1 [file DataSheet_1.docx]

**Supplementary Material**

**Supplementary Tables**

**Supplementary Table 1.** The detailed clinical characteristics of for the patients enrolled in this study

| **Variables** | **TCGA cohort**  **(n=399)** | **GSE32894**  **(n=224)** |
| --- | --- | --- |
| **Age** |  |  |
| <=65 | 158 | 79 |
| >65 | 241 | 145 |
| **Gender** |  |  |
| Female | 105 | 61 |
| Male | 294 | 163 |
| **Tissue** |  |  |
| Normal | 18 | 0 |
| Tumor | 399 | 224 |
| **Grade** |  |  |
| Low | 21 | 129 |
| High | 378 | 95 |
| **Stage** |  |  |
| Ⅰ | 3 | NA |
| Ⅱ | 124 | NA |
| Ⅲ | 139 | NA |
| Ⅳ | 131 | NA |
| Unknown | 2 | NA |
| **Clinical_T** |  |  |
| Ta | 0 | 110 |
| T1 | 8 | 63 |
| T2 | 117 | 43 |
| T3 | 27 | 7 |
| T4 | 11 | 1 |
| Unknown | 236 | 0 |
| **Overall Survival** |  |  |
| Live | 221 | 199 |
| Dead | 178 | 25 |

**Supplementary Table 2.** The primers for qRT-PCR.

| Genes | Forward Primer | Reverse Primer |
| --- | --- | --- |
| CALD1 | 5'-TGGAGGTGAATGCCCAGAAC-3' | 5'-GAAGGCGTTTTTGGCGTCTTT-3' |
| ANXA6 | 5′-AGAGCTACAAGTCCCTCTACG-3′ | 5'- CCCACAATCAACCGTTCAAAC -3' |
| TGFB3 | 5′-GCCTGGAGAAAGCTGCTAAGTA-3′ | 5′-CGTTGTCATACCAGGAAATGAG-3′ |
| β-actin | 5'-GGAGCGAGATCCCTCCAAAAT-3' | 5'-GGCTGTTGTCATACTTCTCATGG-3' |

**Supplementary Table 3.** The peptides of six neoantigens predicted based on TCIA data.

| Patient Barcode | Disease | Neoantigens | Peptide |
| --- | --- | --- | --- |
| TCGA-4Z-AA81 | BLCA | EIF4A2 | EEMPINVADL |
| TCGA-4Z-AA81 | BLCA | EIF4A2 | FYNTTVEEMPI |
| TCGA-4Z-AA81 | BLCA | EIF4A2 | EEMPINVADLI |
| TCGA-C4-A0F7 | BLCA | EIF4A2 | MSGGFADY |
| TCGA-C4-A0F7 | BLCA | EIF4A2 | GGFADYNR |
| TCGA-C4-A0F7 | BLCA | EIF4A2 | MSGGFADYNR |
| TCGA-DK-A6AW | BLCA | EIF4A2 | STFAISILQQL |
| TCGA-K4-A5RH | BLCA | EIF4A2 | VRSGSSRVL |
| TCGA-LT-A5Z6 | BLCA | EIF4A2 | VRNKMQKL |
| TCGA-XF-AAN3 | BLCA | EIF4A2 | GMDPNGVI |
| TCGA-ZF-A9RF | BLCA | EIF4A2 | WLAEKMHAR |
| TCGA-BT-A2LB | BLCA | FANCI | SYKSPVLLL |
| TCGA-BT-A2LB | BLCA | FANCI | LLLRDLSQDI |
| TCGA-BT-A2LB | BLCA | FANCI | SLHVSYKSPVL |
| TCGA-DK-AA75 | BLCA | FANCI | YHFPGPLL |
| TCGA-DK-AA75 | BLCA | FANCI | YHFPGPLLV |
| TCGA-DK-AA75 | BLCA | FANCI | LEAYHFPGPLL |
| TCGA-DK-AA75 | BLCA | FANCI | YHFPGPLLVEL |
| TCGA-G2-A2EO | BLCA | FANCI | LAWYNNTV |
| TCGA-G2-A2EO | BLCA | FANCI | AWYNNTVI |
| TCGA-G2-A2EO | BLCA | FANCI | YNNTVIPL |
| TCGA-G2-A2EO | BLCA | FANCI | CLAWYNNTV |
| TCGA-G2-A2EO | BLCA | FANCI | WYNNTVIPL |
| TCGA-G2-A2EO | BLCA | FANCI | CLAWYNNTVI |
| TCGA-G2-A2EO | BLCA | FANCI | AWYNNTVIPL |
| TCGA-G2-A2EO | BLCA | FANCI | LAWYNNTVIPL |
| TCGA-G2-A2EO | BLCA | FANCI | HFSQSTSI |
| TCGA-G2-A3IE | BLCA | FANCI | CYSFICYV |
| TCGA-G2-A3IE | BLCA | FANCI | SFICYVQNK |
| TCGA-G2-A3IE | BLCA | FANCI | YSFICYVQNK |
| TCGA-G2-A3IE | BLCA | FANCI | CYSFICYVQNK |
| TCGA-UY-A78O | BLCA | FANCI | FMDSYGPK |
| TCGA-UY-A78O | BLCA | FANCI | IFMDSYGPK |
| TCGA-UY-A78O | BLCA | FANCI | FMDSYGPKK |
| TCGA-UY-A78O | BLCA | FANCI | FIFMDSYGPK |
| TCGA-UY-A78O | BLCA | FANCI | IFMDSYGPKK |
| TCGA-UY-A78O | BLCA | FANCI | FMDSYGPKKV |
| TCGA-UY-A78O | BLCA | FANCI | VELGFIFMDSY |
| TCGA-UY-A78O | BLCA | FANCI | GFIFMDSYGPK |
| TCGA-UY-A78O | BLCA | FANCI | FIFMDSYGPKK |
| TCGA-UY-A78O | BLCA | FANCI | FMDSYGPKKVL |
| TCGA-UY-A9PF | BLCA | FANCI | FYEPKPHL |
| TCGA-UY-A9PF | BLCA | FANCI | KPHLLPPL |
| TCGA-UY-A9PF | BLCA | FANCI | FYEPKPHLL |
| TCGA-UY-A9PF | BLCA | FANCI | EPKPHLLPPL |
| TCGA-UY-A9PF | BLCA | FANCI | KPHLLPPLKL |
| TCGA-XF-A9SP | BLCA | FANCI | FSPQFVQM |
| TCGA-XF-A9SP | BLCA | FANCI | SLSKLLEPF |
| TCGA-XF-A9SP | BLCA | FANCI | FSPQFVQML |
| TCGA-XF-A9SP | BLCA | FANCI | TSLSKLLEPF |
| TCGA-XF-A9SP | BLCA | FANCI | KLLEPFSPQF |
| TCGA-XF-A9SP | BLCA | FANCI | EPFSPQFVQM |
| TCGA-XF-A9SP | BLCA | FANCI | EPFSPQFVQML |
| TCGA-XF-AAN0 | BLCA | FANCI | FLIHLCKKSKV |
| TCGA-UY-A9PD | BLCA | OSBPL9 | RLLDSSGSA |
| TCGA-UY-A9PD | BLCA | OSBPL9 | LLDSSGSASV |
| TCGA-UY-A9PD | BLCA | OSBPL9 | RLLDSSGSASV |
| TCGA-UY-A9PD | BLCA | OSBPL9 | LLDSSGSASVL |
| TCGA-XF-A9ST | BLCA | OSBPL9 | LPVGSVLATL |
| TCGA-XF-A9ST | BLCA | OSBPL9 | SLPVGSVLATL |
| TCGA-ZF-A9R7 | BLCA | OSBPL9 | RMVEVVKWY |
| TCGA-ZF-A9R7 | BLCA | OSBPL9 | RMVEVVKWYL |
| TCGA-ZF-A9R7 | BLCA | OSBPL9 | EVVKWYLSAF |
| TCGA-4Z-AA7O | BLCA | SSH3 | TYHKVRLW |
| TCGA-4Z-AA7O | BLCA | SSH3 | FTYHKVRLW |
| TCGA-4Z-AA7O | BLCA | SSH3 | FYPERFTYHKV |
| TCGA-4Z-AA7Y | BLCA | SSH3 | RIFKPICI |
| TCGA-4Z-AA7Y | BLCA | SSH3 | SRIFKPICI |
| TCGA-4Z-AA7Y | BLCA | SSH3 | RIFKPICIQTM |
| TCGA-DK-A2I6 | BLCA | SSH3 | QLQRRQSF |
| TCGA-DK-A2I6 | BLCA | SSH3 | SQLQRRQSF |
| TCGA-DK-A2I6 | BLCA | SSH3 | AVQRRSQLQR |
| TCGA-DK-A2I6 | BLCA | SSH3 | RRSQLQRRQSF |
| TCGA-DK-A6AW | BLCA | SSH3 | RLIEAARA |
| TCGA-DK-A6AW | BLCA | SSH3 | LLPHWKETHRL |
| TCGA-DK-AA77 | BLCA | SSH3 | SQDKTVLL |
| TCGA-DK-AA77 | BLCA | SSH3 | GLSQDKTVLL |
| TCGA-DK-AA77 | BLCA | SSH3 | SQDKTVLLGV |
| TCGA-E7-A85H | BLCA | SSH3 | ARPKPGFLRQL |
| TCGA-FD-A3SS | BLCA | SSH3 | YLGSQWNA |
| TCGA-FD-A3SS | BLCA | SSH3 | SQWNAANL |
| TCGA-FD-A3SS | BLCA | SSH3 | YLGSQWNAA |
| TCGA-FD-A3SS | BLCA | SSH3 | HLYLGSQWNA |
| TCGA-FD-A3SS | BLCA | SSH3 | IFPHLYLGSQW |
| TCGA-FD-A3SS | BLCA | SSH3 | HLYLGSQWNAA |
| TCGA-FD-A3SS | BLCA | SSH3 | YLGSQWNAANL |
| TCGA-FD-A3SS | BLCA | SSH3 | SQWNAANLEEL |
| TCGA-S5-A6DX | BLCA | SSH3 | SVHDSGEEGK |
| TCGA-ZF-A9R7 | BLCA | SSH3 | GLQRRQSF |
| TCGA-ZF-A9R7 | BLCA | SSH3 | QAVQRRSGL |
| TCGA-ZF-A9R7 | BLCA | SSH3 | GLQRRQSFAV |
| TCGA-ZF-A9RC | BLCA | SSH3 | KQYECSLKQA |
| TCGA-ZF-A9RC | BLCA | SSH3 | KQYECSLKQAL |
| TCGA-2F-A9KT | BLCA | ZCCHC8 | SEAGHASSPDF |
| TCGA-4Z-AA89 | BLCA | ZCCHC8 | PMPRYAAR |
| TCGA-4Z-AA89 | BLCA | ZCCHC8 | YAARISEK |
| TCGA-4Z-AA89 | BLCA | ZCCHC8 | CPMPRYAAR |
| TCGA-4Z-AA89 | BLCA | ZCCHC8 | RYAARISEK |
| TCGA-4Z-AA89 | BLCA | ZCCHC8 | YAARISEKR |
| TCGA-4Z-AA89 | BLCA | ZCCHC8 | CPMPRYAARI |
| TCGA-4Z-AA89 | BLCA | ZCCHC8 | RYAARISEKR |
| TCGA-4Z-AA89 | BLCA | ZCCHC8 | YAARISEKRK |
| TCGA-4Z-AA89 | BLCA | ZCCHC8 | RYAARISEKRK |
| TCGA-CU-A3YL | BLCA | ZCCHC8 | SPADMQLDSDM |
| TCGA-DK-A3WW | BLCA | ZCCHC8 | ILTGPSGILV |
| TCGA-DK-AA6P | BLCA | ZCCHC8 | FEFQNMAES |
| TCGA-DK-AA6P | BLCA | ZCCHC8 | FEFQNMAEST |
| TCGA-E7-A7XN | BLCA | ZCCHC8 | KTSEKQTLDK |
| TCGA-GD-A2C5 | BLCA | ZCCHC8 | FSPDSEVTSL |
| TCGA-GD-A2C5 | BLCA | ZCCHC8 | FTKKSEAGHAF |
| TCGA-K4-A54R | BLCA | ZCCHC8 | QRYHAEEVK |
| TCGA-K4-A54R | BLCA | ZCCHC8 | EVKERFGRF |

**Supplementary Figures**


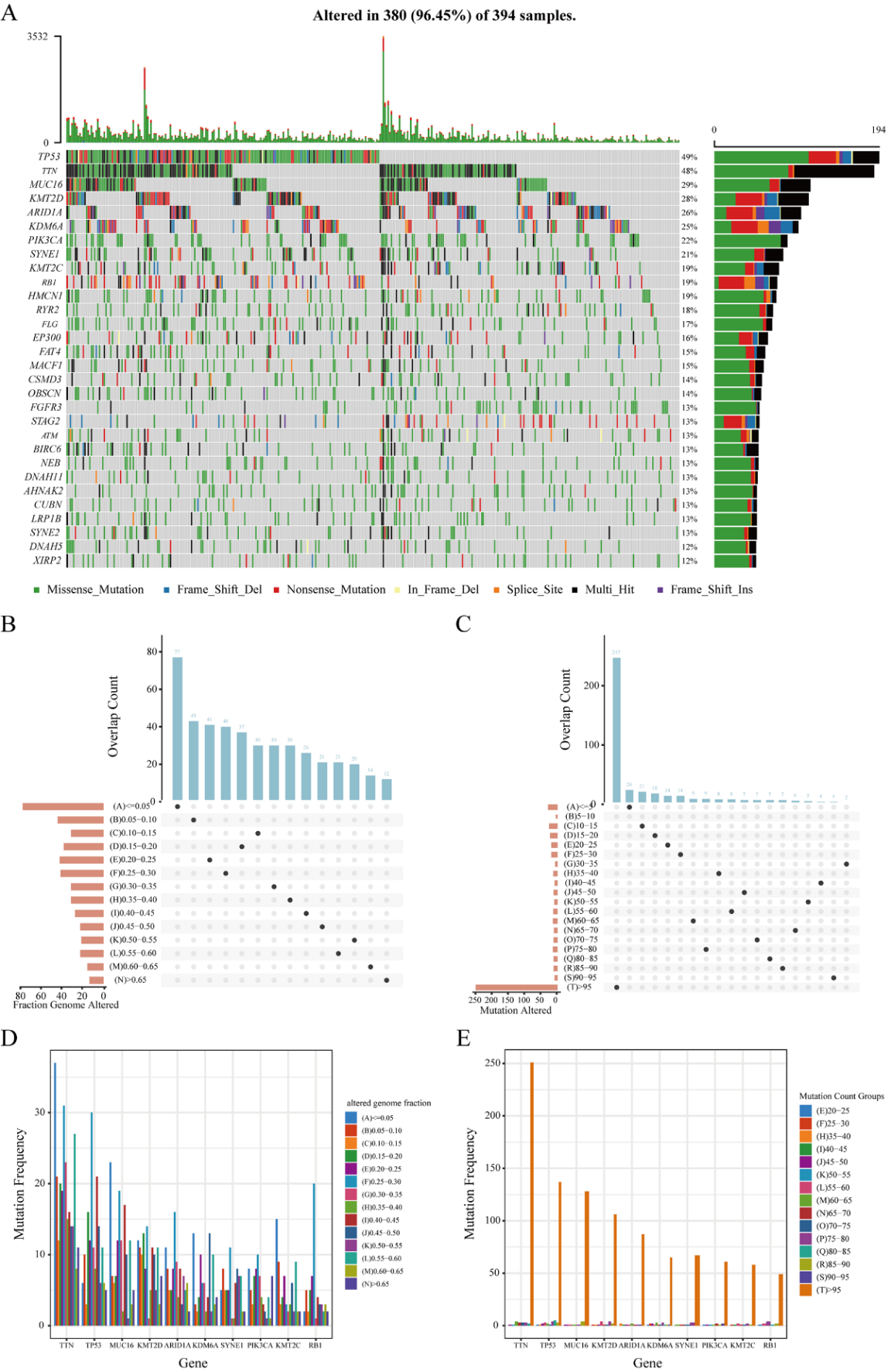


**Supplementary Figure 1.** Identification of potential tumour antigens in BLCA. (A) Mutation status of the top 20 mutated genes in patients with BLCA. (B-E) Identification of potential tumour-specific antigens of BLCA. Samples overlapping in altered genome fraction (B) and mutation count (C) groups. Genes with the highest frequency in altered genome fraction (D) and mutation count (E) groups.


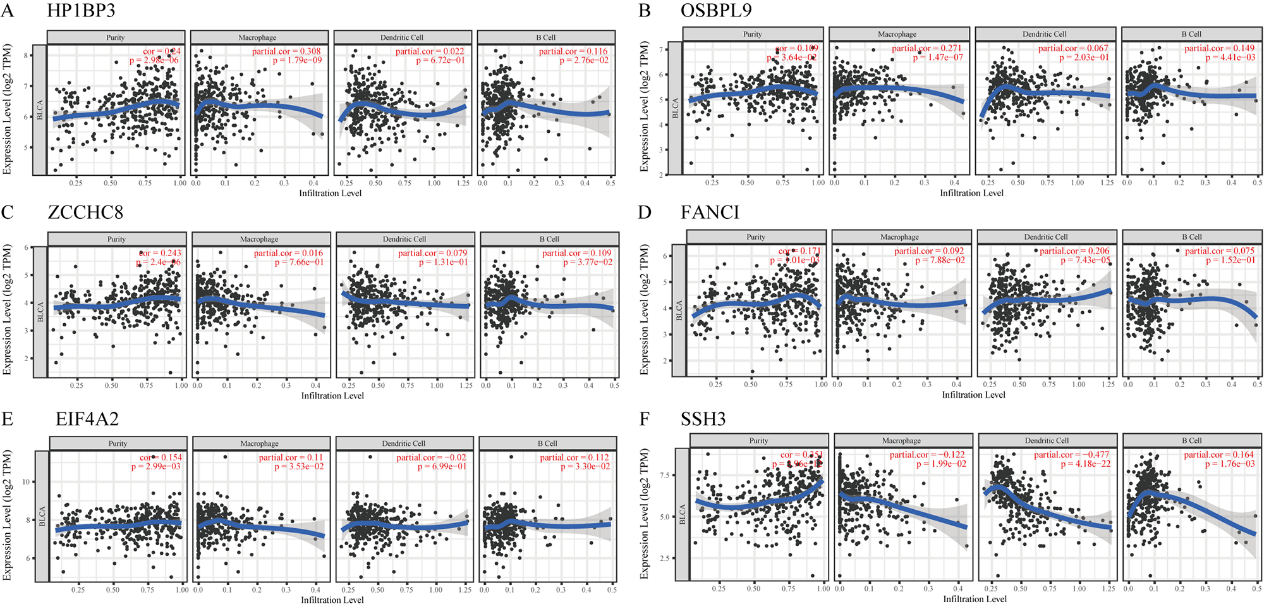


**Supplementary Figure 2.** Identification of tumour antigens associated with antigen-presenting cells. Correlation of (A) HP1BP3, (B) OSBPL9, (C) ZCCHC8, (D) FANCI, (E) EIF4A2 and (F) SSH3 with the infiltration of macrophages, dendritic cells and B cells.

**
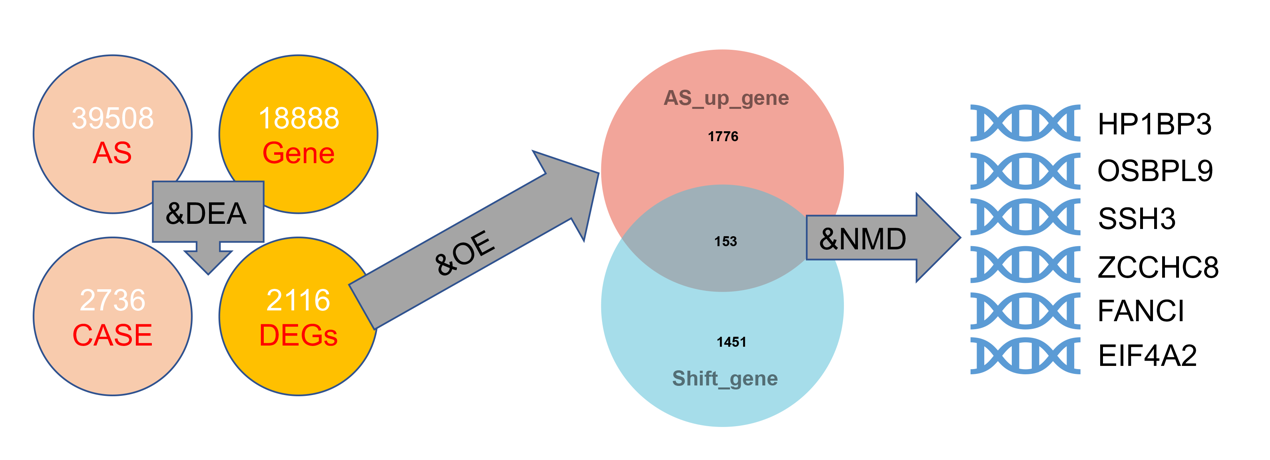
**

**Supplementary Figure 3.** A flow diagram of 6 predicted tumor antigens from candidate overexpressed genes, genes with AS aberration and frameshift mutations, and NMD-related genes.


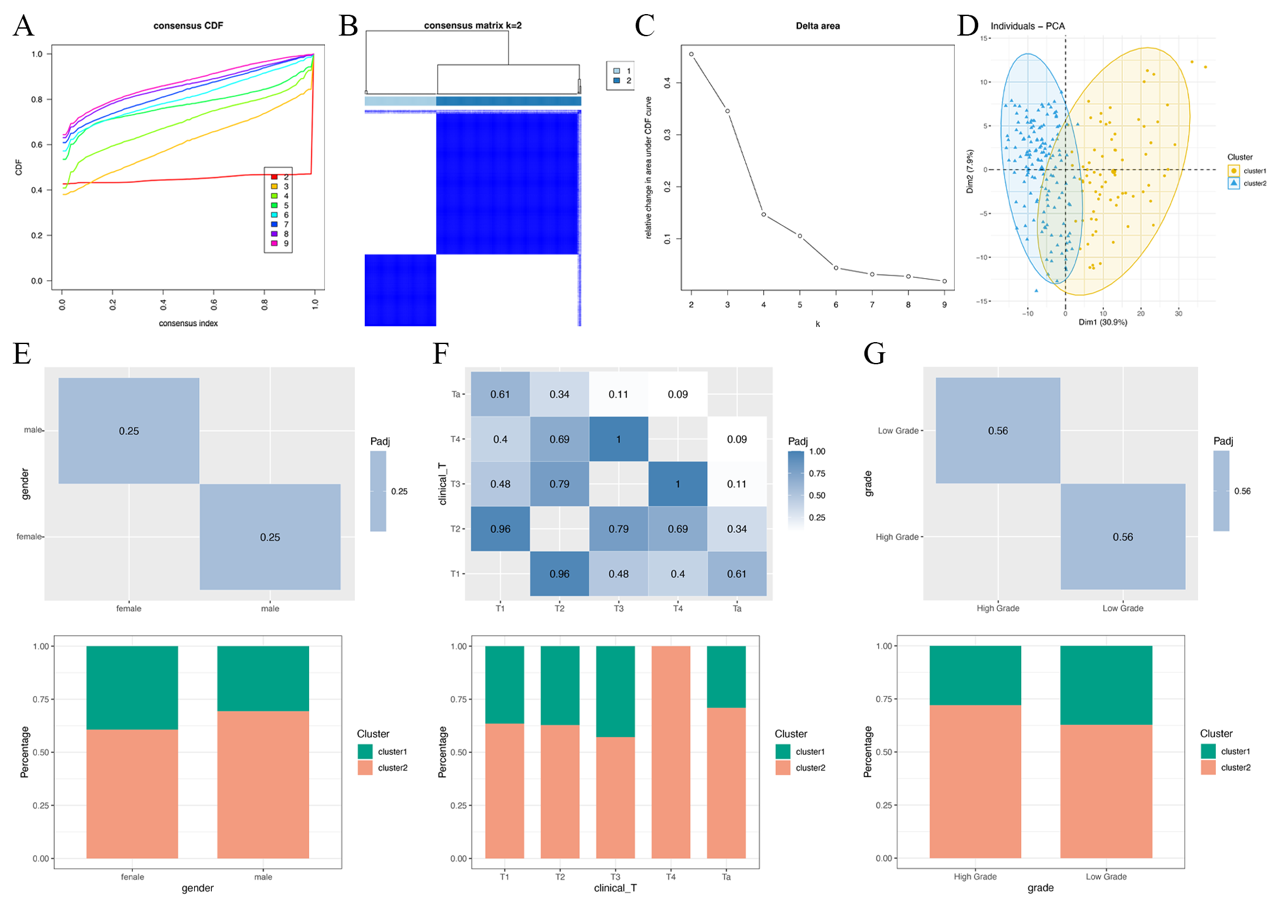


**Supplementary Figure 4.** Validation of immune clusters of BLCA in the GSE32894 cohort. (A) Cumulative distribution function curve and (B) delta area of immune-related genes in TCGA cohort. (C) Sample clustering heatmap. (D) Principal component analysis demonstrating two distinct clusters reflecting immune status. (E–G) Distribution of IC1–2 based on (E) sex, (F) clinical T stage, and (G) grade.


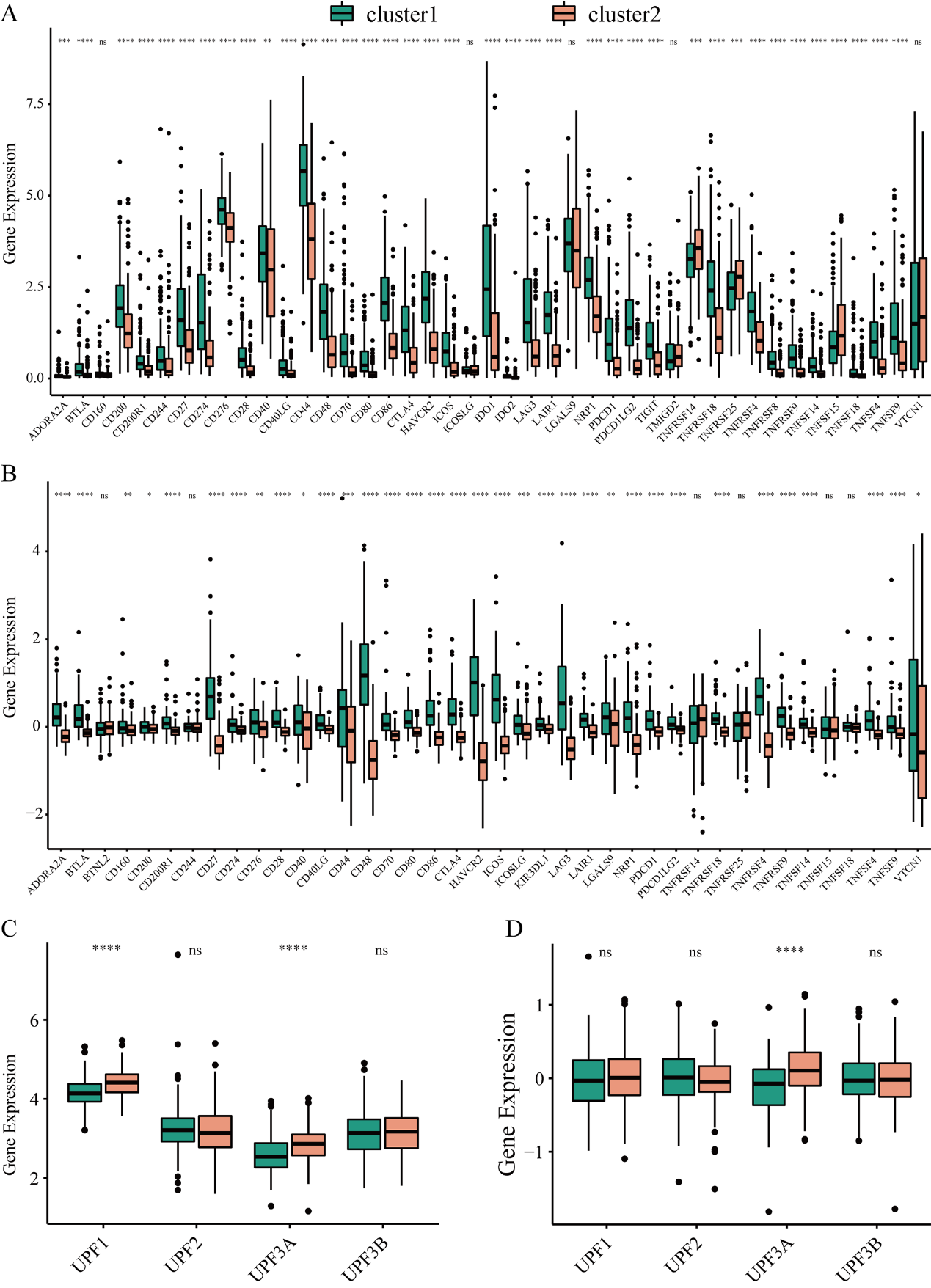


**Supplementary Figure 5.** Association of immune clusters with ICPs and NMD factors. (A-B) Differences in the expression of ICPs between the two immune clusters in (A) TCGA and (B) GSE32894 cohorts. (C-D) Differences in the expression of NMD factors between the two immune clusters in (C) TCGA and (D) GSE32894 cohorts. * P < 0.05, ** P < 0.01, *** P < 0.001, **** P < 0.0001; ns: not significant.


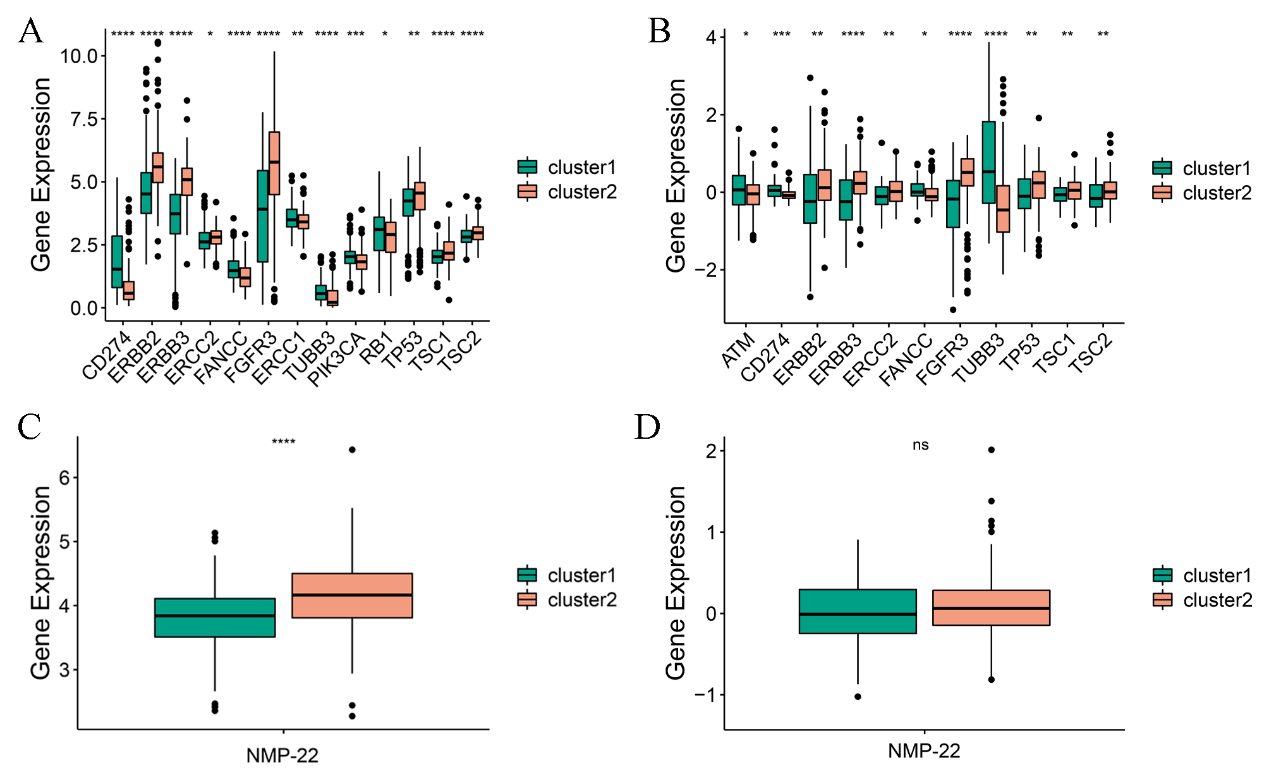


**Supplementary Figure 6.** Association of immune clusters with tumour markers. (A-B) Expression of tumour markers in BLCA immune clusters based on CGI data in (A) TCGA and (B) GSE32894 cohorts. (C-D) Expression of NMP-22 in BLCA immune clusters in (C) TCGA and (D) GSE32894 cohorts. * P < 0.05, ** P < 0.01, *** P < 0.001, **** P < 0.0001; ns: not significant.


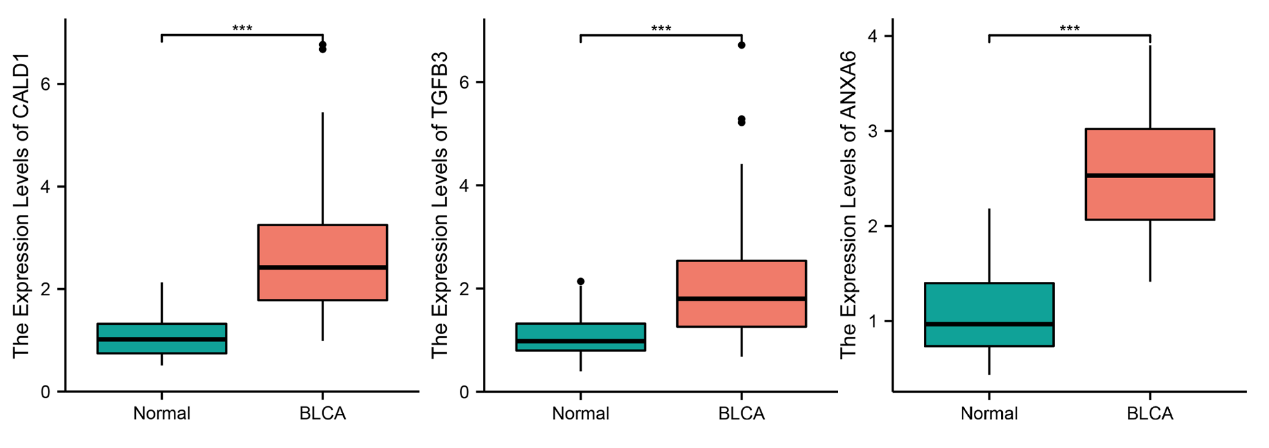


**Supplementary Figure 7.** RT-qPCR was used to detect the relative mRNA levels of CALD1, TGFB3 and ANXA6 in BLCA and adjacent normal tissues. *** P < 0.001.
